# Pointwise Encoding Time Reduction With Radial Acquisition MRI-Derived Osteochondral Thickness Ratio for Assessing Early Knee Osteoarthritis: A Prospective Case-Control Study

**DOI:** 10.7759/cureus.116678

**Published:** 2026-09-21

**Authors:** Hao Ma, Qianyi Zhao, Jiaxin Yin, Yuhao Zhao, Jiawen Tang, Jiatuo Zhang, Shizhou Tang, Fengzhang Zhu, Zheng Yan, Xianglin Zhang

**Affiliations:** 1 Radiology, The First Affiliated Hospital of Jinzhou Medical University, Jinzhou, CHN; 2 Radiology, The First Affiliated Hospital of Dalian Medical University, Dalian, CHN; 3 Translational Medicine, The First Affiliated Hospital of Jinzhou Medical University, Jinzhou, CHN; 4 Ultrasound Medicine, Jinhua People's Hospital, Jinhua, CHN; 5 Clinical Biospecimen, The First Affiliated Hospital of Jinzhou Medical University, Jinzhou, CHN; 6 Radiology, Nanyang Central Hospital, Nanyang, CHN

**Keywords:** cartilage thickness, early knee osteoarthritis, osteochondral unit, petra mri, radiology

## Abstract

Background: Early knee osteoarthritis (EKOA) may present with persistent symptoms despite mild radiographic changes. This prospective case-control study evaluated whether a pointwise encoding time reduction with radial acquisition (PETRA)-derived osteochondral thickness ratio (F ratio) could characterize structural differences between patients with EKOA and healthy controls.

Methods: Participants underwent weight-bearing radiography, conventional magnetic resonance imaging (MRI), and 3.0 T PETRA MRI. The F ratio was calculated as the sum of mean superficial cartilage, deep cartilage, and osteochondral junction thicknesses divided by mean subchondral bone plate thickness. We compared F ratios and individual layer measures between 59 participants with EKOA and 54 healthy controls, assessed within-sample discrimination using receiver operating characteristic analysis with bootstrap internal validation, and evaluated inter-reader and intra-reader reproducibility in 23 participants.

Results: The median F ratio was lower in the EKOA group than in healthy controls (1.998 (interquartile range, 1.604-2.567) versus 2.692 (2.401-3.000); P < 0.001). The adjusted geometric mean ratio was 0.776 (95% confidence interval, 0.703-0.857; P < 0.001). Among knees with measurable superficial cartilage, superficial cartilage thickness was lower in EKOA (0.944 ± 0.298 mm versus 1.331 ± 0.285 mm; P < 0.001), whereas subchondral bone plate thickness was greater (1.521 ± 0.468 mm versus 1.320 ± 0.270 mm; false-discovery-rate-adjusted q = 0.019). Superficial cartilage was non-identifiable in 16 of 59 EKOA knees (27.1%) and in no controls (P < 0.001). The apparent area under the curve for EKOA discrimination was 0.766 (bootstrap 95% confidence interval, 0.674-0.851); the optimism-corrected area under the curve was 0.765. Inter-reader and intra-reader intraclass correlation coefficients for the F ratio were 0.988 and 0.993, respectively.

Conclusions: The PETRA MRI-derived F ratio was lower in EKOA and showed high measurement reproducibility with moderate within-sample discrimination. This measure may serve as a supplementary descriptor of osteochondral structural phenotype in patients with persistent knee pain and mild radiographic change. External and longitudinal validation is required before clinical diagnostic use.

## Introduction

Knee osteoarthritis (KOA) is a major cause of chronic pain and activity limitation in middle-aged and older adults. The Global Burden of Disease Study shows that the number of people living with osteoarthritis continues to increase, with the knee as the most commonly affected site; population aging and high body mass index (BMI) further increase this burden [1]. Current KOA management focuses on symptom relief, preservation of function, and modification of treatable risk factors. Identifying early structural abnormalities can improve patient assessment and support research on disease progression [2]. Early intervention may improve treatment response and long-term quality of life. Patients with early KOA (EKOA) commonly have persistent knee pain despite inconspicuous radiographic findings. This discordance between symptoms and radiographic changes complicates early assessment. Definitions based on symptoms, clinical signs, and imaging also remain inconsistent across studies, limiting comparisons and standardized identification of early disease [3]. The Chinese guideline for diagnosis and non-surgical treatment of early-stage knee osteoarthritis (2024 edition) recommends integrating clinical manifestations with imaging findings and provides the framework for case definition in this study [4].

The osteochondral unit provides the structural link between articular cartilage degeneration and subchondral bone change. Cartilage is classified histologically by its extracellular matrix as hyaline, elastic, or fibrocartilage. Hyaline cartilage is rich in type II collagen and proteoglycans and provides low-friction, compressive load-bearing surfaces. Elastic cartilage contains additional elastic fibers, whereas fibrocartilage contains more type I collagen and less proteoglycan and is highly resistant to tension and compression. Hyaline cartilage is present at most synovial joint surfaces; the meniscus is fibrocartilage [5]. Deep cartilage, calcified cartilage, the osteochondral junction (OCJ), and the subchondral bone plate are anatomically continuous and jointly transmit load during the development of osteoarthritis. Their short T2 relaxation times cause the signal to decay rapidly at conventional magnetic resonance imaging (MRI) echo times (TEs), which limits visualization of local osteochondral layers [6]. In a study of 143 knees, three-dimensional ultrashort TE (UTE) MRI findings of calcified cartilage defects and thinning, together with subchondral bone plate abnormalities, were associated with cartilage degeneration [7]. In EKOA, cartilage T2 and OCJ UTE-T2* also show regional alterations, indicating that different tissues and anatomic sites provide complementary information [8]. Evaluating the osteochondral unit as an integrated structure, while supplementing conventional whole-joint assessment with OCJ and subchondral bone plate information, may therefore provide a more complete structural phenotype of EKOA.

Pointwise encoding time reduction with radial acquisition (PETRA) combines radial acquisition of peripheral k-space with single-point encoding of central k-space, enabling UTEs and three-dimensional near-isotropic imaging on clinical MRI systems [9]. In vivo studies have shown that PETRA can capture short-T2 signal in the meniscus and can help distinguish normal, degenerated, and torn tissue [10]. Ex vivo PETRA with fat suppression has further demonstrated laminar non-calcified cartilage structure and identified cartilage injury against histologic reference [11]. These studies establish a technical basis for PETRA evaluation of short-T2 tissues in the knee, but current evidence primarily addresses focal lesion detection or signal-parameter analysis. Systematic quantification of the relative thicknesses of cartilage, OCJ, and subchondral bone plate in a uniformly defined EKOA population remains limited.

Accordingly, this study compared PETRA-derived osteochondral unit features in 59 patients with EKOA and 54 healthy controls using a clinical 3.0 T MRI system. We measured the apparent thicknesses of superficial cartilage, deep cartilage, the OCJ, and the subchondral bone plate, then calculated the ratio of cartilage-plus-OCJ thickness to subchondral bone plate thickness (F ratio). We assessed its association with EKOA, measurement reproducibility, and within-sample discrimination, and explored associations with pain and radiographic grade. The study aimed to provide an interpretable candidate quantitative marker for osteochondral structural phenotyping in symptomatic individuals with mild radiographic change and to inform future clinical stratification, longitudinal follow-up, and disease-progression research.

## Materials and methods

Study design and ethics

This was a single-center, prospective, observational case-control study. Participants were consecutively recruited at the Department of Radiology, First Affiliated Hospital of Jinzhou Medical University, from December 17, 2025, to May 18, 2026. The protocol was approved by the Ethics Committee of the First Affiliated Hospital of Jinzhou Medical University (approval No. 2026LL-KY-098). All participants provided written informed consent.

Participants

Inclusion and Exclusion Criteria

The EKOA group included participants aged 40 years or older. All participants underwent clinical assessment, weight-bearing knee radiography, and MRI. EKOA was determined by an integrated assessment according to the Chinese guideline for diagnosis and non-surgical treatment of early-stage knee osteoarthritis (2024 edition), with Kellgren-Lawrence (K-L) grade restricted to 0 to 2 [4]. Participants had not received medication or surgical treatment for osteoarthritis during the month before enrollment.

Inclusion criteria for the EKOA group were age 40 years or older; persistent knee pain or functional limitation; completion of weight-bearing knee radiography, conventional MRI, and PETRA; K-L grade 0-2; and complete clinical and imaging data permitting prespecified measurements. Healthy controls were recruited during the same period and were required to have no persistent knee pain or functional limitation, K-L grade 0-1 on weight-bearing radiography, and the same imaging examinations. EKOA was determined jointly by a clinician and a senior radiologist on the basis of recorded symptoms, signs, weight-bearing radiographs, and conventional MRI.

Healthy controls were volunteers recruited during the same period who had no persistent knee pain or functional limitation and a K-L grade of 0 or 1 on weight-bearing radiography. They met the same exclusion criteria as the EKOA group.

Exclusion criteria for both groups were inflammatory or metabolic arthropathy, including rheumatoid arthritis and gout; K-L grade greater than 2; acute knee fracture, acute meniscal injury, or other substantial trauma that could affect image assessment within the preceding six months; previous knee replacement or other knee surgery; MRI-incompatible metal implants or other MRI contraindications; claustrophobia or inability to tolerate MRI; and active rheumatic disease.

Exclusion criteria for both groups were inflammatory or metabolic arthropathy, including rheumatoid arthritis and gout; K-L grade > 2; acute fracture, acute meniscal injury, or other substantial trauma that could affect measurement; previous knee surgery or intra-articular fracture; tumor or infection; MRI contraindication; inadequate image quality; or missing key clinical or imaging data. Data selection followed prespecified quality-control rules for completeness, study side, denominator validity, and formula consistency; participants were not excluded on the basis of analytic results or outlyingness.

Selection of the Study Knee and Case Determination

One knee was included per participant. In the EKOA group, the symptomatic knee was selected; when both knees met eligibility criteria, one was selected according to a prespecified random rule. One knee was randomly selected in healthy controls.

Only one knee was included per participant. For unilateral eligibility in EKOA, the symptomatic side was selected; when both knees were eligible, one side was selected by a prespecified random rule. One side was randomly selected for healthy controls. The clinician and senior radiologist independently assigned K-L grades, whole-organ MRI score (WORMS), and final groups, resolving disagreements by consensus.

K-L grade [12], WORMS, and final group assignment were independently determined by a physician and a senior radiologist, with disagreements resolved by consensus. WORMS provides a standardized evaluation of multistructural abnormalities on conventional knee MRI [13]. Final grouping was based on clinical manifestations, weight-bearing radiographs, and conventional MRI findings. PETRA quantitative measures did not contribute to group assignment.

Sample Size

Before enrollment, sample size was estimated in a receiver operating characteristic (ROC) area under the curve (AUC) testing framework: alternative AUC A = 0.80, null AUC A0 = 0.50, two-sided alpha = 0.05, power 1-beta = 0.80, and approximately 1:1 cases to controls. The target standard error (SE) was $\mathrm{SE} = (A - A0)/[z(1 - \mathrm{alpha}/2) + z(1 - \mathrm{beta})]$, and the Hanley-McNeil approximate variance $\mathrm{Var}(A) = {A(1 - A) + (n1 - 1)[Q1 - A^{2}] + (n0 - 1)[Q2 - A^{2}]}/(n1n0)$ was solved iteratively, where *Q1 = A/(2 - A)* and $Q2 = 2A^{2}/(1 + A)$. Calculations used a reproducible Python 3.12.14 script (Python Software Foundation, Fredericksburg, VA, USA). With prespecified covariate-adjusted analyses and precision requirements, the protocol conservatively targeted 50 per group plus approximately 10% for scan failure or incomplete data, planning 55 per group. Consecutive convenience sampling was used. Of 114 people screened, one healthy control did not complete MRI because of claustrophobia; 59 participants with EKOA and 54 healthy controls were included.

Imaging assessment

Radiography and Conventional MRI

All participants underwent weight-bearing knee radiography and MRI. Weight-bearing radiography and conventional MRI were used for K-L grading, integrated EKOA determination, WORMS assessment, and anatomic localization for PETRA measurements.

MRI Acquisition

MRI was performed using a 3.0 T system (MAGNETOM Vida; Siemens Healthineers, Erlangen, Germany) with an 18-channel knee coil (TxRx_Knee_18). Sequence parameters are shown in Table 1. PETRA used an axial three-dimensional acquisition: repetition time (TR)/TE 3.37/0.07 ms, field of view (FOV) 332 x 332 mm, matrix 320 x 320, acquired voxel size approximately 1.041 x 1.041 x 1.041 mm, flip angle 3 degrees, number of excitations (NEX) 1, and acquisition time 2 min 19 s. Full parameters are provided in Table 1.

**Table 1:** MRI acquisition parameters. This table presents the scan protocol. TR: repetition time; TE: echo time; FOV: field of view; ETL: echo train length; NEX: number of excitations; GRAPPA: generalized autocalibrating partially parallel acquisitions; PETRA: pointwise encoding time reduction with radial acquisition; FS: fat suppression; NA: not applicable or not recorded; 3D: three-dimensional; MRI: magnetic resonance imaging

Sequence	Plane	TR (ms)	TE (ms)	FOV readout (mm)	FOV phase (mm)	Matrix readout	Matrix phase	Voxel x (mm)	Voxel y (mm)	Voxel z (mm)	Slice gap (mm)	Flip angle (degrees)	ETL	NEX	GRAPPA	Acquisition time
PD-TSE FS	Sagittal	2,450	22	180	180	384	384	0.469	0.469	3.5	4.2	120	8	2	NA	2 min 29 s
T1-TSE	Sagittal	550	12	160	160	384	320	NA	NA	3.0	0-0.3	NA	3-5	1-2	2	2 min 20 s
PD-TSE FS	Coronal	4,130	40	160	160	384	384	0.42	0.42	3.0	3.3	130	13	1	NA	1 min 48 s
T2-TSE FS	Axial	6,400	63	160	160	320	320	0.5	0.5	3.0	3.3	136	15	1	NA	1 min 30 s
3D PETRA	Axial	3.37	0.07	332	332	320	320	1.041	1.041	Approximately 1.041	NA	3	NA	1	NA	2 min 19 s

PETRA Sequence

PETRA combines radial acquisition of peripheral k-space with single-point encoding of central k-space to capture short-T2 tissue signal [9]. We used an axial three-dimensional PETRA sequence with an acquired isotropic voxel size of approximately 1.041 mm and a nominal spatial resolution of approximately 1 x 1 x 1 mm. The sequence added 2 minutes and 19 s to the conventional knee MRI examination and supported multiplanar reconstruction (MPR) in arbitrary orientations.

Image postprocessing and quantitative measurement

PETRA source images were imported into 3D Slicer version 5.12.3 (Brigham and Women's Hospital, Harvard Medical School, Boston, MA, USA) for MPR. Readers localized the femoral condylar weight-bearing region in three orthogonal planes and adjusted the reconstruction plane so that measurements were approximately perpendicular to the local articular surface. PETRA images were displayed with grayscale inversion to improve visual contrast between adjacent layers. Conventional MRI was used to confirm anatomic location and adjacent structures, while PETRA was used to evaluate cartilage structure (Figure 1).

**Figure 1:**
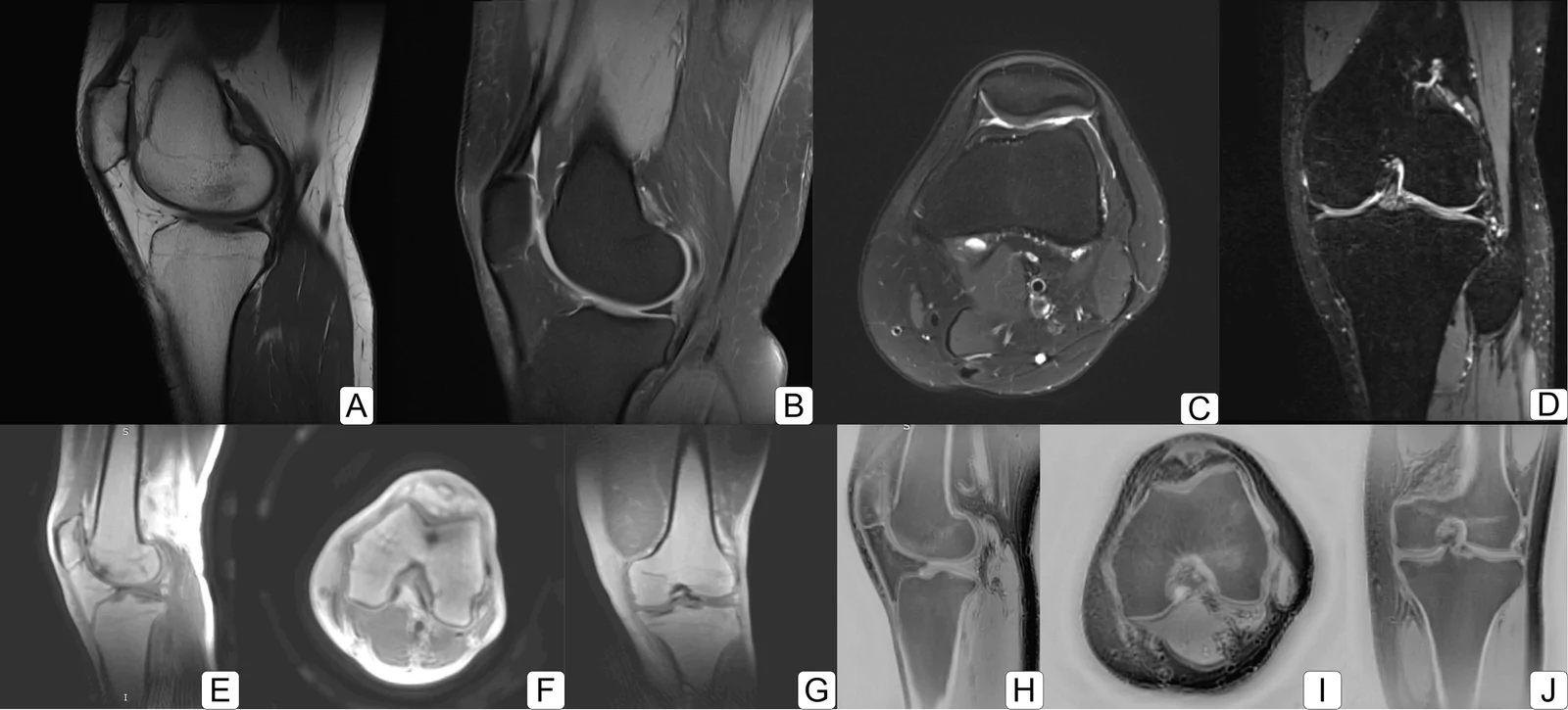
Conventional and PETRA MRI of the knee. (A) Sagittal T1-weighted image. (B) Sagittal proton density-weighted image. (C) Axial T2-weighted image. (D) Coronal proton density-weighted image. (E) Sagittal PETRA image. (F) Axial PETRA image. (G) Coronal PETRA image. (H) Grayscale-inverted sagittal PETRA image. (I) Grayscale-inverted axial PETRA image. (J) Grayscale-inverted coronal PETRA image. All clinical MR images are original de-identified images from participants in this study; PETRA MPR and grayscale inversion were performed in 3D Slicer 5.12.3 (Brigham and Women's Hospital, Harvard Medical School, Boston, MA, USA); panels were assembled in draw.io (Wiesbaden, Germany). PETRA: pointwise encoding time reduction with radial acquisition; MRI: magnetic resonance imaging; MPR: multiplanar reconstruction

PETRA source images were imported into 3D Slicer 5.12.3 for MPR. The weight-bearing region of the femoral condyle was localized in sagittal, coronal, and axial planes, with the reconstruction plane made as perpendicular as possible to the local articular surface. A consistent grayscale-inverted display was used to identify the continuous signal bands and interfaces of superficial cartilage, deep cartilage, the OCJ, and the subchondral bone plate.

Readers identified the imaging bands corresponding to superficial cartilage, deep cartilage, the OCJ, and the subchondral bone plate according to continuous signal bands and their interfaces on grayscale-inverted PETRA images (Figure 2). The layer names were used for anatomic localization; the measured values represent imaging-based apparent thickness at the acquisition resolution. A previous PETRA-fat-suppressed study with histologic comparison provided a reference for laminar visualization of non-calcified cartilage [11].

**Figure 2:**
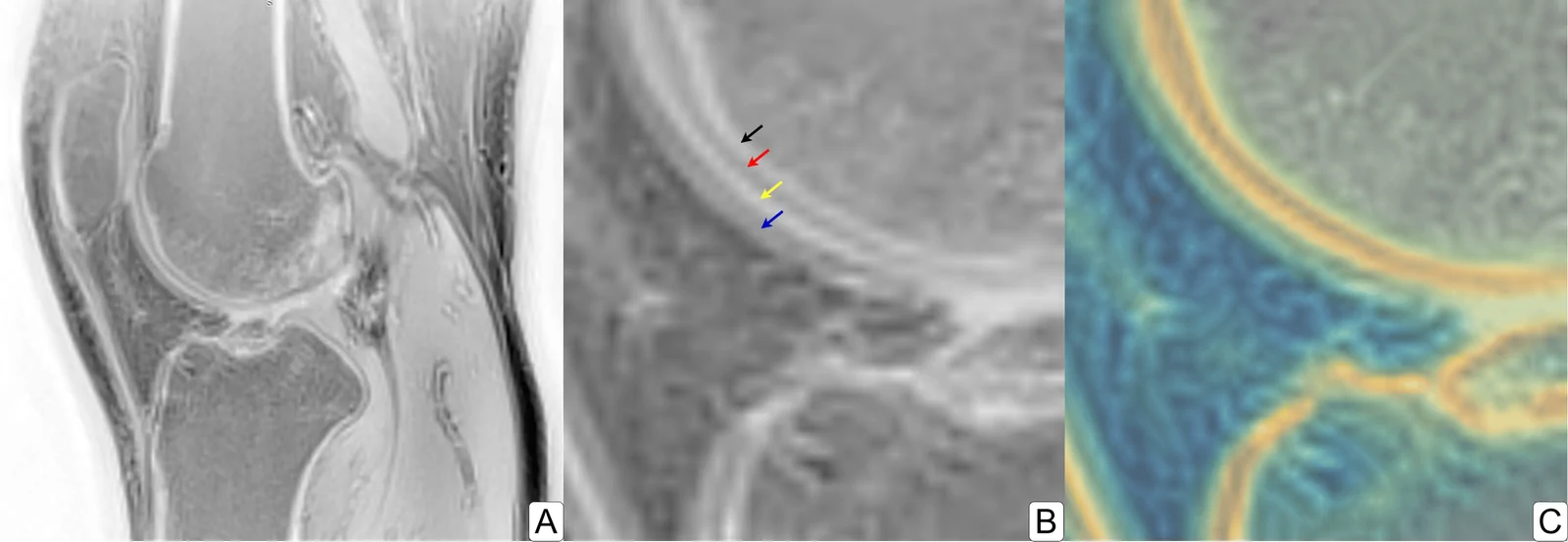
PETRA layer-band identification and measurement of the osteochondral unit. (A) Grayscale-inverted sagittal PETRA image of a healthy knee. (B) Magnified view of femoral cartilage showing laminar structure: the blue arrow indicates superficial cartilage; yellow arrow, deep cartilage; red arrow, the OCJ; and black arrow, the subchondral bone plate. (C) Pseudocolor image of femoral cartilage, in which the laminar pattern is more conspicuous. Images are original de-identified PETRA MR images from this study; MPR, grayscale inversion, and measurement annotations were performed in 3D Slicer 5.12.3 (Brigham and Women's Hospital, Harvard Medical School, Boston, MA, USA), and panels were assembled in draw.io (Wiesbaden, Germany). PETRA: pointwise encoding time reduction with radial acquisition; MPR: multiplanar reconstruction; MR: magnetic resonance; OCJ: osteochondral junction

At prespecified medial, central, and lateral locations, electronic calipers were placed along the local articular-surface normal to measure superficial-cartilage, deep-cartilage, OCJ, and subchondral-bone-plate thicknesses. Each layer was measured once at each location, and the arithmetic mean across the three locations was used as the layer thickness for the study knee, in millimeters. When superficial cartilage was non-identifiable on PETRA, thickness was recorded as 0 mm at all three locations and included in the F ratio according to the prespecified rule; this value represents an imaging-defined absent structure rather than missing-value imputation.

When the superficial cartilage band could not be identified on PETRA, thickness at all three locations was recorded as 0 mm according to a prespecified rule and included in the F ratio calculation. This zero value was an analytic code for imaging non-identifiability, not imputation of a missing value. Analyses of superficial cartilage thickness as a continuous variable included only participants in whom the layer remained identifiable and measurable. Superficial cartilage non-identifiability was analyzed separately as a binary imaging feature (Figure 3).

**Figure 3:**
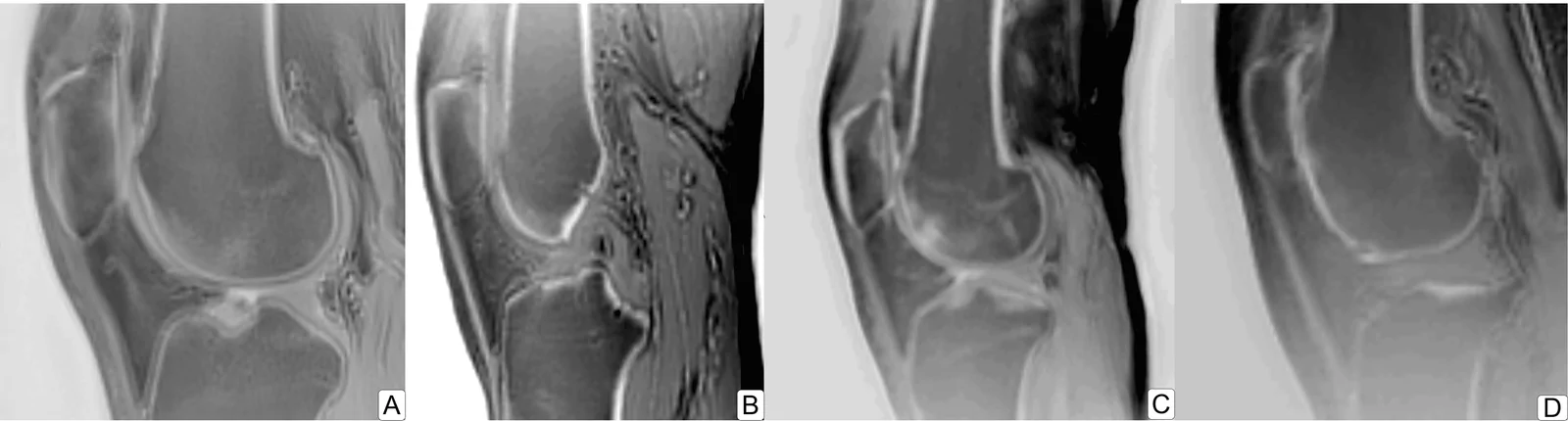
Representative grayscale-inverted PETRA appearances of superficial cartilage morphology in patients with EKOA. (A) Swelling of cartilage at the anterior margin of the distal femur with patchy high signal in the subchondral bone. (B) Superficial cartilage thinning, slight thickening of deep cartilage and the OCJ, and patchy high signal in the subchondral bone. (C) Superficial cartilage thinning with focal loss and surface irregularity. (D) Absence of superficial cartilage, injury to deep cartilage, irregular OCJ architecture, and widening of the subchondral bone plate. Images are original de-identified PETRA MR images from this study; MPR and grayscale inversion were performed in 3D Slicer 5.12.3 (Brigham and Women's Hospital, Harvard Medical School, Boston, MA, USA), and panels were assembled in draw.io (Wiesbaden, Germany). PETRA: pointwise encoding time reduction with radial acquisition; MR: magnetic resonance; EKOA: early knee osteoarthritis; OCJ: osteochondral junction; MPR: multiplanar reconstruction

Outcomes

The primary imaging outcome was the prespecified dimensionless osteochondral thickness ratio, F, which describes the relative thickness of the cartilage-plus-OCJ side and the subchondral bone plate:

$\displaystyle F = \frac{\overline{T}_{\mathrm{superficial\ cartilage}} + \overline{T}_{\mathrm{deep\ cartilage}} + \overline{T}_{\mathrm{OCJ}}}{\overline{T}_{\mathrm{subchondral\ bone\ plate}}}$

Formula: *F = (S + D + C)/B = N/B*, where S is mean superficial-cartilage thickness, D is mean deep-cartilage thickness, C is mean OCJ thickness, B is mean subchondral-bone-plate thickness, and *N = S + D + C*.

Secondary imaging measures included mean thicknesses of superficial cartilage, deep cartilage, the OCJ, and the subchondral bone plate; superficial cartilage non-identifiability; OCJ morphology; marginal micro-osteophytes; meniscal findings; ligament-insertion findings; and focal low-signal lesions. Focal low-signal lesions were compared between groups as present or absent.

Pain was assessed with the Western Ontario and McMaster Universities Osteoarthritis Index (WOMAC) pain subscale [14]. We used five Likert items scored from 0 to 4, yielding a total score of 0 to 20, with higher scores indicating more severe pain. Symptom duration was also recorded for participants with EKOA.

The WOMAC pain subscale uses five Likert items scored 0-4, for a total of 0-20; higher scores indicate more severe pain. The original scale is cited in reference [14]. The original K-L grading system is cited in reference [12], and WORMS in reference [13].

Blinding and measurement reproducibility

Before quantitative measurement, all images were anonymized and recoded. Readers were blinded to group assignment, clinical information, WOMAC score, K-L grade, WORMS results, and the other reader's measurements.

Inter-reader agreement for continuous measures was assessed using a two-way random-effects, absolute-agreement, single-measure intraclass correlation coefficient (ICC(A,1)/ICC(2,1)); intra-reader agreement was assessed using a two-way mixed-effects, absolute-agreement, single-measure ICC(A,1). We report 95% confidence intervals (CIs) for all ICCs [15]. Bland-Altman analysis was used to estimate mean bias and 95% limits of agreement and to assess proportional bias [16]. For categorical and ordinal imaging features, unweighted or quadratic-weighted Cohen kappa, respectively, was calculated with 95% CIs derived from 10,000 participant-level bootstrap resamples; raw agreement was also reported.

Categorical imaging features were interpreted according to prespecified criteria. Inter-reader and intra-reader reproducibility were assessed with Cohen kappa, with raw agreement also reported.

Statistical analysis

Statistical analyses were performed using Python 3.12.14 and R 4.6.1 (R Foundation for Statistical Computing, Vienna, Austria). Except for deterministic exact/Firth analyses in R, the resampling random seed was fixed at 20260904. All tests were two-sided; P < 0.05 was considered statistically significant, and for multiple comparisons, the threshold was Benjamini-Hochberg-adjusted q < 0.05. Primary analyses did not impute missing values and report the actual analytic sample sizes.

Normality of continuous variables was assessed with the Shapiro-Wilk test and summarized as mean ± standard deviation or median and interquartile range, as appropriate. Categorical variables were summarized as number and percentage. Baseline continuous variables were compared with Welch t-tests and categorical variables with chi-squared or Fisher exact tests.

The primary analysis compared F ratios between groups using the Mann-Whitney U test, with rank-biserial correlation and stratified bootstrap 95% CIs reported. Robustness was assessed with Welch t-tests, Hedges g, and HC3 heteroscedasticity-consistent linear regression. The basic regression model adjusted for age, sex, and side; the BMI-adjusted model additionally included BMI. F ratio was log-transformed, and regression coefficients were exponentiated to report geometric mean ratios (GMRs).

Between-group F ratio comparisons primarily used the Mann-Whitney U test, with rank-biserial correlation and 95% CIs from 20,000 group-stratified bootstrap resamples; Welch t tests, Hedges g, and HC3 heteroscedasticity-robust linear regression were supplementary. Base regression models adjusted for age, sex, and study side, and extended models additionally adjusted for BMI. Absent superficial structure used two-sided Fisher exact testing; because controls had zero events, Firth penalized-likelihood regression was a sensitivity analysis.

Individual layer thicknesses were compared between groups with Mann-Whitney U tests, and effect sizes were reported. Superficial cartilage thickness analysis included only participants in whom that layer was measurable. Superficial cartilage, deep cartilage, OCJ, and subchondral bone plate thicknesses formed a family of four tests and were false discovery rate (FDR)-corrected within that family. Cartilage-plus-OCJ thickness was reported descriptively as a component of the F ratio and was not included in this testing family.

Superficial cartilage non-identifiability was compared with a two-sided Fisher exact test. Ordered OCJ morphology, meniscal, ligament-insertion, and micro-osteophyte measures were compared with Mann-Whitney U tests; focal low-signal lesions were compared as binary outcomes. These five measures formed the morphology testing family and were FDR-corrected within that family.

Spearman rank correlations assessed the relation between F ratio and K-L grade, with results reported separately by group. In the EKOA group, we further assessed associations of F ratio with WOMAC pain score, symptom duration, and superficial cartilage morphology grade, and the association between K-L grade and superficial cartilage morphology grade. The multivariable model for F ratio and WOMAC pain adjusted for age, sex, BMI, and K-L grade. Symptom duration was transformed as log(1 + duration) for a supplementary HC3 heteroscedasticity-consistent linear regression. These clinical association analyses were exploratory and were not included in the primary multiplicity control.

ROC analysis assessed the ability of negative F ratio to distinguish EKOA from healthy control status. We calculated AUC and bootstrap 95% CIs. A within-sample cutoff was selected using the Youden index, with sensitivity and specificity calculated at that cutoff. Two thousand bootstrap resamples estimated AUC optimism, and optimism-corrected AUC and Youden index were reported. This analysis estimated within-sample discrimination, and the cutoff is presented as exploratory.

ROC analysis used -F as the score in the EKOA direction and reported apparent AUC and 95% CIs from 20,000 stratified bootstrap resamples. The Youden cutoff, sensitivity, and specificity were within-sample exploratory results only; the cutoff was reselected in each bootstrap sample, and optimism was estimated using 2,000 bootstrap resamples. No training/test split was used.

To examine the components of the F ratio difference, the sum of superficial cartilage, deep cartilage, and OCJ thickness was denoted N, and subchondral bone plate thickness was denoted B. Based on log(F) = log(N) - log(B), models adjusted for age, sex, side, and BMI separately estimated group effects on log(F), log(N), and log(B). The relative contributions of the N and B sides to the between-group difference in log(F) were calculated with bootstrap 95% CIs. This post hoc decomposition describes the mathematical components of the ratio.

Sensitivity analyses excluded observations with extremely thin subchondral bone plates, used Huber robust regression, and performed leave-one-out analyses to evaluate the influence of extreme denominators and model specification. Linear mixed-effects models also evaluated medial, central, and lateral locations, including group, location, and group-by-location interaction terms with a participant-level random intercept to account for correlated measurements within each knee. Sensitivity and location analyses were post hoc and exploratory; unadjusted P-values are reported.

Huber robust regression was used only to test stability of the F ratio group coefficient. Separate Huber models for log(F), log(N), and log(B) yield different weights and cannot ensure beta_F = beta_N - beta_B; numerator/denominator contributions were therefore not calculated from Huber results. Component decomposition is reported only for HC3/OLS models with the same covariate set.

## Results

Participant characteristics and baseline findings

Shapiro-Wilk normality results are presented in Table 2. Of 18 within-group tests, seven indicated departure from normality (P < 0.05), primarily for deep cartilage, OCJ, subchondral bone plate, and cartilage-plus-OCJ thickness, as well as WOMAC pain score and symptom duration in the EKOA group. Age, BMI, F ratio, and superficial cartilage thickness did not show clear departure from normality in either group. The prespecified Mann-Whitney U test was used for the between-group comparison of F ratio (Table 2).

**Table 2:** Shapiro-Wilk normality tests for continuous variables. All tests were two-sided; P < 0.05 indicates statistical evidence of departure from normality. EKOA: early knee osteoarthritis; BMI: body mass index; OCJ: osteochondral junction; WOMAC: Western Ontario and McMaster Universities Osteoarthritis Index

Variable	Group	n	W statistic	P-value	Conclusion
Age	EKOA	59	0.976	0.293	No evidence of departure from normality
Age	Healthy control	54	0.957	0.051	No evidence of departure from normality
BMI	EKOA	59	0.975	0.275	No evidence of departure from normality
BMI	Healthy control	54	0.989	0.894	No evidence of departure from normality
F ratio	EKOA	59	0.977	0.34	No evidence of departure from normality
F ratio	Healthy control	54	0.989	0.915	No evidence of departure from normality
Superficial cartilage thickness	EKOA	43	0.955	0.092	No evidence of departure from normality
Superficial cartilage thickness	Healthy control	54	0.975	0.322	No evidence of departure from normality
Deep cartilage thickness	EKOA	59	0.789	<0.001	Evidence of departure from normality
Deep cartilage thickness	Healthy control	54	0.914	<0.001	Evidence of departure from normality
OCJ thickness	EKOA	59	0.874	<0.001	Evidence of departure from normality
OCJ thickness	Healthy control	54	0.975	0.332	No evidence of departure from normality
Subchondral bone plate thickness	EKOA	59	0.81	<0.001	Evidence of departure from normality
Subchondral bone plate thickness	Healthy control	54	0.969	0.169	No evidence of departure from normality
Total osteochondral-unit thickness	EKOA	59	0.929	0.002	Evidence of departure from normality
Total osteochondral-unit thickness	Healthy control	54	0.987	0.803	No evidence of departure from normality
WOMAC pain	EKOA	59	0.956	0.034	Evidence of departure from normality
Symptom duration	EKOA	59	0.918	<0.001	Evidence of departure from normality

A total of 114 individuals were screened. One healthy control did not complete MRI because of claustrophobia, leaving 113 participants: 59 with EKOA and 54 healthy controls. Mean ages were 58.39 ± 7.54 and 55.81 ± 7.36 years, respectively (P = 0.069). Women accounted for 50.8% (30/59) and 55.6% (30/54) of the groups (P = 0.616), and left knees accounted for 44.1% (26/59) and 46.3% (25/54), respectively (P = 0.812). BMI was higher in the EKOA group (25.03 ± 2.90 kg/m² vs 23.85 ± 2.76 kg/m², P = 0.030). In the EKOA group, 11 (18.6%), 40 (67.8%), and eight (13.6%) participants had K-L grades 0, 1, and 2, respectively. In healthy controls, 26 (48.1%) and 28 (51.9%) participants had grades 0 and 1. The EKOA group had a mean WOMAC pain score of 8.12 ± 5.34, a median of 8.00, and an interquartile range of 3.50 to 11.00. Mean symptom duration was 4.49 ± 2.36 months, with a median of 4.00 months and an interquartile range of 3.00 to 6.00 months (Table 3).

**Table 3:** Baseline characteristics of the study participants. Percentages use the total number in each group as the denominator. Age and BMI were tested using two-sided Welch t-tests; sex and study side using two-sided Pearson chi-squared tests; K-L grade, WOMAC, and symptom duration are reported descriptively. SD: standard deviation; Q1/Q3: first/third quartile; SMD: standardized mean difference; NA: not applicable; EKOA: early knee osteoarthritis; BMI: body mass index; WOMAC: Western Ontario and McMaster Universities Osteoarthritis Index; K-L: Kellgren-Lawrence

Continuous variables																	
Variable	Group		n		Mean	SD		Median		Q1		Q3	Test		P-value		SMD
Age (years)	EKOA		59		58.39	7.54		60.0		54.0		63.0	Welch t		0.069		0.346
Age (years)	Healthy control		54		55.81	7.36		55.0		50.0		62.0	Welch t		0.069		0.346
BMI (kg/m²)	EKOA		59		25.03	2.9		25.0		23.0		27.3	Welch t		0.03		0.415
BMI (kg/m²)	Healthy control		54		23.85	2.76		23.95		22.2		25.98	Welch t		0.03		0.415
WOMAC pain (0-20)	EKOA		59		8.12	5.34		8.0		3.5		11.0	Descriptive		NA		NA
Symptom duration (months)	EKOA		59		4.49	2.36		4.0		3.0		6.0	Descriptive		NA		NA
Categorical variables																	
Variable		Level		Group			n		%		Test			P-value		SMD	
Sex		Female		EKOA			30		50.8		χ²			0.616		-0.094	
Sex		Female		Healthy control			30		55.6		χ²			0.616		-0.094	
Sex		Male		EKOA			29		49.2		χ²			0.616		-0.094	
Sex		Male		Healthy control			24		44.4		χ²			0.616		-0.094	
Study side		Right		EKOA			33		55.9		χ²			0.812		0.045	
Study side		Right		Healthy control			29		53.7		χ²			0.812		0.045	
Study side		Left		EKOA			26		44.1		χ²			0.812		0.045	
Study side		Left		Healthy control			25		46.3		χ²			0.812		0.045	
K-L grade		0		EKOA			11		18.6		Descriptive			NA		NA	
K-L grade		0		Healthy control			26		48.1		Descriptive			NA		NA	
K-L grade		1		EKOA			40		67.8		Descriptive			NA		NA	
K-L grade		1		Healthy control			28		51.9		Descriptive			NA		NA	
K-L grade		2		EKOA			8		13.6		Descriptive			NA		NA	
K-L grade		2		Healthy control			0		0.0		Descriptive			NA		NA	

Key case-control comparisons are shown in Table 3. Differences in age (Welch t-test P = 0.069, standardized mean difference (SMD) = 0.346), sex (chi-squared P = 0.616, SMD = -0.094), and study side (chi-squared P = 0.812, SMD = 0.045) were not significant; BMI was higher in EKOA (25.03 ± 2.90 vs 23.85 ± 2.76 kg/m²; Welch t-test P = 0.030, SMD = 0.415).

F ratio and sensitivity analyses

The F ratio was lower in the EKOA group than in healthy controls, with medians of 1.998 (interquartile range, 1.604 to 2.567) and 2.692 (interquartile range, 2.401 to 3.000), respectively (Mann-Whitney U test, P < 0.001). The rank-biserial correlation was -0.532 (95% CI -0.701 to -0.351). After adjustment for age, sex, side, and BMI, the group regression coefficient was -0.531 (95% CI -0.748 to -0.314; P < 0.001), and the GMR from the log-transformed model was 0.776 (95% CI 0.703 to 0.857; P < 0.001).

The F ratio was lower in EKOA than in healthy controls (median 1.998 vs 2.692; Mann-Whitney U P < 0.001; rank-biserial correlation -0.532, 95% CI -0.701 to -0.351; Figure 4 and Table 4). The group coefficient in the BMI-adjusted HC3 model was -0.531 (95% CI -0.748 to -0.314, P < 0.001; Table 5). Huber robust regression yielded beta_F = -0.249 as an F ratio sensitivity result only; Table 6 no longer reports invalid Huber beta_N, beta_B, or contribution percentages.

**Figure 4:**
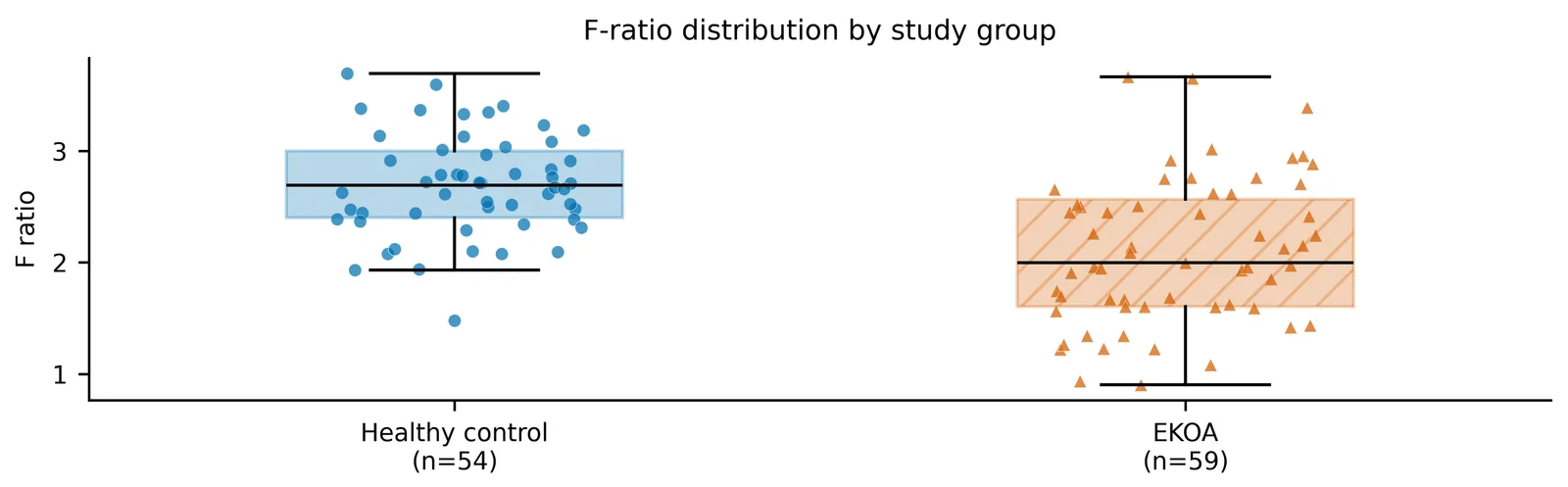
Distribution of the F ratio in the EKOA and healthy control groups. EKOA: early knee osteoarthritis

**Table 4:** F ratio and osteochondral thickness parameters. Thicknesses are in mm; all tests were two-sided. M-W: Mann-Whitney U test; mean differences were estimated in a Welch t-test framework; effect-size CIs used 20,000 stratified bootstrap resamples. The F ratio was the prespecified primary measure and was not included in the layer-thickness FDR family; therefore, q is NA. BH-FDR: Benjamini-Hochberg false discovery rate; CI: 95% confidence interval; SD: standard deviation; EKOA: early knee osteoarthritis; OCJ: osteochondral junction

Descriptive statistics															
Parameter			Group			n				Mean			SD		
F ratio			EKOA			59				2.099			0.658		
F ratio			Healthy control			54				2.692			0.458		
Superficial cartilage			EKOA			43				0.944			0.298		
Superficial cartilage			Healthy control			54				1.331			0.285		
Deep cartilage			EKOA			59				1.268			0.517		
Deep cartilage			Healthy control			54				1.122			0.295		
OCJ			EKOA			59				1.087			0.388		
OCJ			Healthy control			54				1.016			0.192		
Subchondral bone plate			EKOA			59				1.521			0.468		
Subchondral bone plate			Healthy control			54				1.32			0.27		
Total osteochondral-unit thickness			EKOA			59				3.043			0.88		
Total osteochondral-unit thickness			Healthy control			54				3.468			0.543		
Between-group effects															
Parameter	Rank-biserial correlation	CI lower bound		CI upper bound	M-W P		Mean difference	CI lower bound	CI upper bound		Hedges g	CI lower bound		CI upper bound	BH-FDR q
F ratio	-0.532	-0.701		-0.351	<0.001		-0.593	-0.803	-0.383		-1.031	-1.484		-0.656	NA
Superficial cartilage	-0.675	-0.828		-0.498	<0.001		-0.386	-0.505	-0.268		-1.318	-1.847		-0.882	<0.001
Deep cartilage	0.201	-0.017		0.417	0.066		0.146	-0.01	0.302		0.341	0.0		0.649	0.083
OCJ	0.074	-0.142		0.292	0.499		0.072	-0.042	0.185		0.229	-0.129		0.542	0.499
Subchondral bone plate	0.277	0.067		0.478	0.011		0.201	0.059	0.342		0.516	0.214		0.817	0.019
Total osteochondral-unit thickness	-0.43	-0.617		-0.233	<0.001		-0.425	-0.696	-0.154		-0.571	-1.049		-0.198	<0.001

**Table 5:** HC3 robust regression models. Base models adjusted for age, sex, and study side; BMI-adjusted models additionally adjusted for BMI. The target coefficient in the WOMAC model was the F ratio; that in the symptom-duration model was log(1 + duration). In the log(F) model, the EKOA/control GMR was 0.776 (95% CI 0.703-0.857). All P values were two-sided. HC3: heteroscedasticity-consistent covariance; SE: standard error; GMR: geometric mean ratio; BMI: body mass index; EKOA: early knee osteoarthritis; CI: confidence interval; OCJ: osteochondral junction; WOMAC: Western Ontario and McMaster Universities Osteoarthritis Index

Outcome	Model	Group/target coefficient	SE	CI lower bound	CI upper bound	P-value	n	R²	Adjusted R²
Superficial cartilage	Base HC3	-0.373	0.061	-0.493	-0.253	<0.001	97	0.365	0.338
Superficial cartilage	BMI-adjusted HC3	-0.353	0.062	-0.474	-0.231	<0.001	97	0.387	0.353
Deep cartilage	Base HC3	0.122	0.079	-0.034	0.277	0.125	113	0.056	0.021
Deep cartilage	BMI-adjusted HC3	0.123	0.083	-0.039	0.286	0.138	113	0.056	0.012
OCJ	Base HC3	0.057	0.056	-0.053	0.167	0.313	113	0.046	0.01
OCJ	BMI-adjusted HC3	0.05	0.059	-0.066	0.166	0.402	113	0.048	0.004
Subchondral bone plate	Base HC3	0.164	0.068	0.03	0.298	0.016	113	0.151	0.12
Subchondral bone plate	BMI-adjusted HC3	0.151	0.072	0.01	0.292	0.036	113	0.158	0.118
Total osteochondral-unit thickness	Base HC3	-0.454	0.135	-0.718	-0.19	<0.001	113	0.108	0.075
Total osteochondral-unit thickness	BMI-adjusted HC3	-0.433	0.145	-0.718	-0.149	0.003	113	0.112	0.071
F ratio	Base HC3	-0.555	0.106	-0.763	-0.347	<0.001	113	0.24	0.212
F ratio	BMI-adjusted HC3	-0.531	0.111	-0.748	-0.314	<0.001	113	0.248	0.213
log(F)	BMI-adjusted HC3	-0.253	0.051	-0.353	-0.154	<0.001	113	0.261	0.227
WOMAC pain	EKOA clinical HC3	-1.035	1.096	-3.182	1.113	0.345	59	0.235	0.163
F ratio	EKOA symptom-duration HC3	0.192	0.269	-0.335	0.719	0.475	59	0.064	-0.024

**Table 6:** Sensitivity analyses for the F ratio. beta_F = beta_N - beta_B; contribution percentages were calculated as |beta_N|/[|beta_N| + |beta_B|] and |beta_B|/[|beta_N| + |beta_B|]. The Huber model was used only to assess robustness of the F ratio; fitting N and B separately yields different robust weights, so Huber beta_N, beta_B, and contribution percentages are not reported. All models adjusted for age, sex, and study side and, unless stated otherwise, additionally for BMI. GMR: geometric mean ratio; N: superficial cartilage + deep cartilage + OCJ; B: subchondral bone plate; HC3: heteroscedasticity-consistent covariance estimator; EKOA: early knee osteoarthritis; BMI: body mass index; OCJ: osteochondral junction

Robustness of the F ratio									
Analysis		n			βF			EKOA/control GMR	
Primary HC3/OLS		113			-0.253			0.776	
Without BMI adjustment		113			-0.266			0.766	
Excluding B ≤ 1.04 mm		97			-0.244			0.783	
Huber robust regression		113			-0.249			0.78	
Component decomposition in harmonized HC3/OLS models									
Analysis	n		βF	βN		βB	Numerator contribution (%)		Denominator contribution (%)
Primary HC3/OLS	113		-0.253	-0.162		0.091	63.9		36.1
Without BMI adjustment	113		-0.266	-0.165		0.1	62.2		37.8
Excluding B ≤ 1.04 mm	97		-0.244	-0.179		0.065	73.2		26.8

Welch testing, Hedges g, covariate adjustment, exclusion of extremely thin bone plates, Huber robust regression, and leave-one-out analysis all showed the same direction of difference, supporting the robustness of the lower F ratio in EKOA. Analyses excluding extremely thin plates, Huber regression, and leave-one-out analyses are interpreted as post hoc exploratory findings (Figure 4; Table 6).

The primary F ratio analysis included all 59 participants with EKOA and 54 healthy controls. Superficial cartilage was coded as 0 mm according to the non-identifiability rule in 16 participants with EKOA. Analysis of superficial cartilage thickness alone used the measurable subset of 43 participants with EKOA and 54 healthy controls.

Thicknesses of individual osteochondral unit layers

Except for the analysis restricted to participants with measurable superficial cartilage, all thickness analyses included 59 participants with EKOA and 54 healthy controls. Among measurable cases, superficial cartilage was thinner in EKOA than in controls (0.944 ± 0.298 mm, n = 43 vs 1.331 ± 0.285 mm, n = 54; Mann-Whitney U test, P < 0.001; rank-biserial correlation, -0.675, 95% CI -0.828 to -0.498; Hedges g, -1.318, 95% CI -1.847 to -0.882; FDR-adjusted q < 0.001). Group coefficients in the basic and BMI-adjusted models were -0.373 mm (95% CI -0.493 to -0.253; P < 0.001) and -0.353 mm (95% CI -0.474 to -0.231; P < 0.001), respectively.

Layer-thickness comparisons are shown in Table 4: superficial cartilage was thinner among measurable participants (n = 43 vs 54; P < 0.001; q < 0.001), the subchondral bone plate was thicker (n = 59 vs 54; P = 0.011; q = 0.019), and total osteochondral-unit thickness was lower (P < 0.001; q < 0.001). Differences in deep cartilage and OCJ did not pass the FDR threshold (q = 0.083 and 0.499). Corresponding adjusted models are in Table 5.

Subchondral bone plate thickness was 1.521 ± 0.468 mm in EKOA and 1.320 ± 0.270 mm in controls (unadjusted P = 0.011; FDR-adjusted q = 0.019). Cartilage-plus-OCJ thickness was lower in EKOA (3.043 ± 0.880 mm vs 3.468 ± 0.543 mm, P < 0.001). This composite measure is reported descriptively as a component of the F ratio and was not included in the layer-thickness testing family.

Deep cartilage thickness was 1.268 ± 0.517 mm in EKOA and 1.122 ± 0.295 mm in controls (P = 0.066; q = 0.083). OCJ thickness was 1.087 ± 0.388 mm and 1.016 ± 0.192 mm, respectively (P = 0.499; q = 0.499). Neither comparison reached the prespecified FDR significance threshold (Tables 4, 5).

Superficial cartilage non-identifiability and morphological findings

Superficial cartilage was non-identifiable on PETRA in 16 of 59 participants with EKOA (27.1%) and in none of 54 healthy controls (two-sided Fisher exact test, P < 0.001). Within the morphology testing family, OCJ morphology, meniscal score, and ligament-insertion score were higher in EKOA (q < 0.001, q = 0.008, and q = 0.041, respectively). The prevalence of focal low-signal lesions also differed between groups (q < 0.001), whereas marginal micro-osteophytes did not meet the FDR threshold (q = 0.077) (Table 7).

**Table 7:** Morphological findings. Percentages use the total number in the respective group as the denominator. All tests were two-sided. The primary inference for absent superficial structure was the Fisher exact test; Firth regression was used only as a zero-event sensitivity analysis and was not included in the morphology FDR family. OR: odds ratio; CI: 95% confidence interval; BH-FDR: Benjamini-Hochberg false discovery rate; NA: not applicable; OCJ: osteochondral junction; EKOA: early knee osteoarthritis

Between-group inference											
Finding	Test		Effect measure	Estimate		CI lower bound		CI upper bound	P-value		BH-FDR q
OCJ morphology	Ordinal Mann-Whitney		Rank-biserial correlation	0.466		0.311		0.611	<0.001		<0.001
Marginal micro-osteophyte	Pearson chi square		OR	2.126		0.913		4.949	0.077		0.077
Meniscal score	Ordinal Mann-Whitney		Rank-biserial correlation	0.29		0.099		0.47	0.005		0.008
Ligament insertion score	Ordinal Mann-Whitney		Rank-biserial correlation	0.215		0.025		0.402	0.033		0.041
Any low-signal lesion	Pearson chi square		OR	31.5		10.502		94.48	<0.001		<0.001
Absent superficial structure	Fisher exact		P-value	<0.001		NA		NA	<0.001		NA
Absent superficial structure	Firth sensitivity analysis		OR	41.345		5.324		5,327.235	<0.001		NA
Frequency distribution											
Finding		Level			Group		n			%	
Superficial cartilage morphology		Normal			EKOA		19			32.2	
Superficial cartilage morphology		Rough/indistinct margin			EKOA		24			40.7	
Superficial cartilage morphology		Absent structure			EKOA		16			27.1	
Absent superficial structure		Yes			Healthy control		0			0.0	
Absent superficial structure		Yes			EKOA		16			27.1	
Low-signal lesion		None			EKOA		14			23.7	
Low-signal lesion		Patchy			EKOA		31			52.5	
Low-signal lesion		Linear			EKOA		14			23.7	

Absent superficial-cartilage structure occurred in 16/59 EKOA knees (27.1%) and 0/54 controls (0.0%); two-sided Fisher exact P < 0.001. The Firth sensitivity analysis gave odds ratio (OR) = 41.34 (penalized profile-likelihood 95% CI 5.32-5,327.24, P < 0.001). Between-group statistics and FDR-adjusted results for OCJ morphology, meniscus, ligament insertion, and low-signal lesions are in Table 7.

Associations of F ratio with K-L grade, pain, and symptom duration

The Spearman correlation between F ratio and K-L grade was -0.121 in the EKOA group (95% CI -0.400 to 0.182; P = 0.361) and -0.350 in healthy controls (95% CI -0.579 to -0.084; P = 0.010). In EKOA, the correlation between F ratio and WOMAC pain score was -0.106 (95% CI -0.368 to 0.173; P = 0.424); after adjustment for age, sex, BMI, and K-L grade, the regression coefficient was -1.035 (95% CI -3.182 to 1.113; P = 0.345). The correlation between F ratio and symptom duration was 0.119 (95% CI -0.143 to 0.368; P = 0.368). In the supplementary HC3 model, the coefficient for log(1 + symptom duration) was 0.192 (95% CI -0.335 to 0.719; P = 0.475). The CIs for these EKOA associations crossed zero (Figures 5, 6; Table 8).

**Figure 5:**
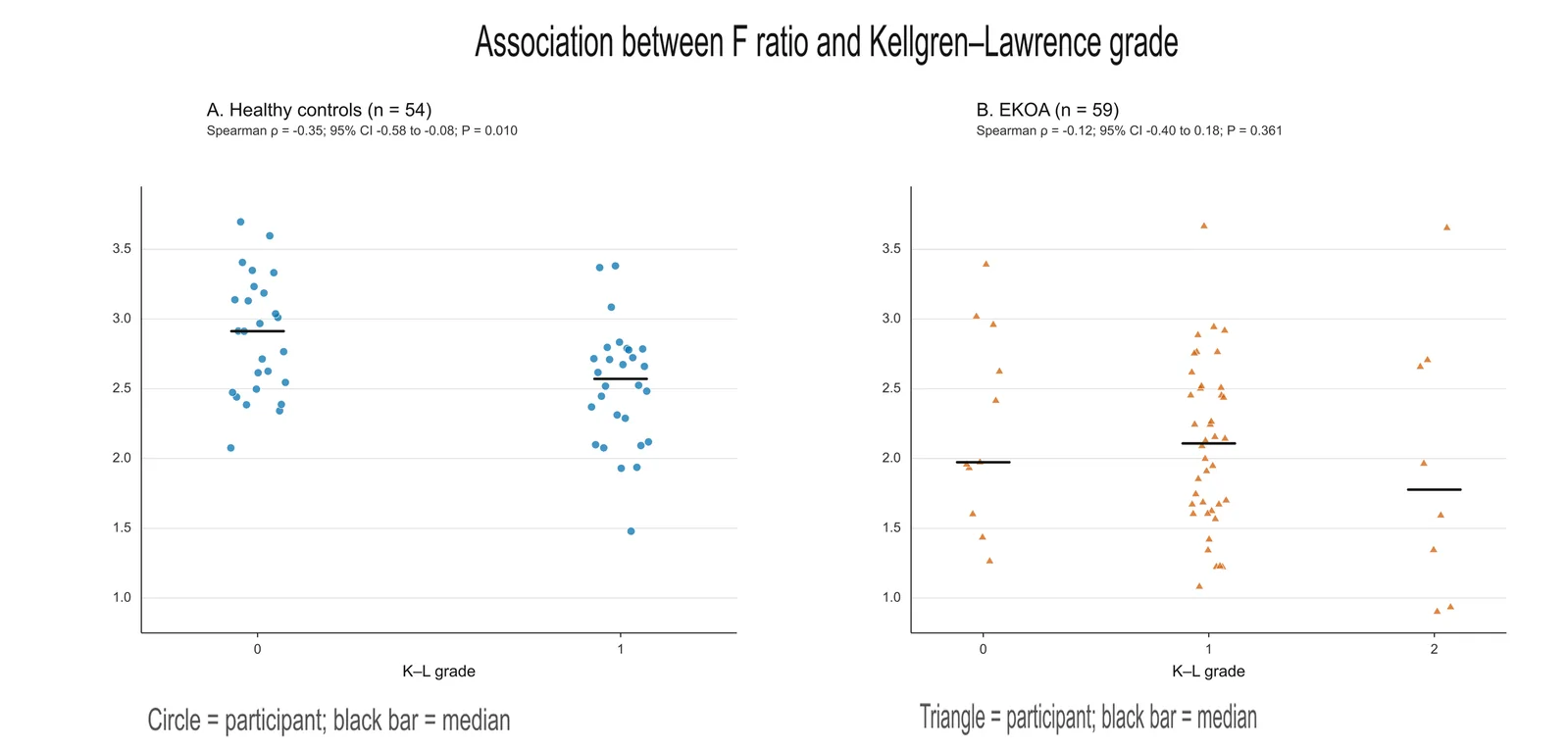
Distribution of the F ratio by K-L grade in healthy controls (A) and participants with EKOA (B). Horizontal lines indicate group medians; displayed Spearman coefficients summarize the association within each group. K-L: Kellgren-Lawrence; EKOA: early knee osteoarthritis; CI: confidence interval

**Figure 6:**
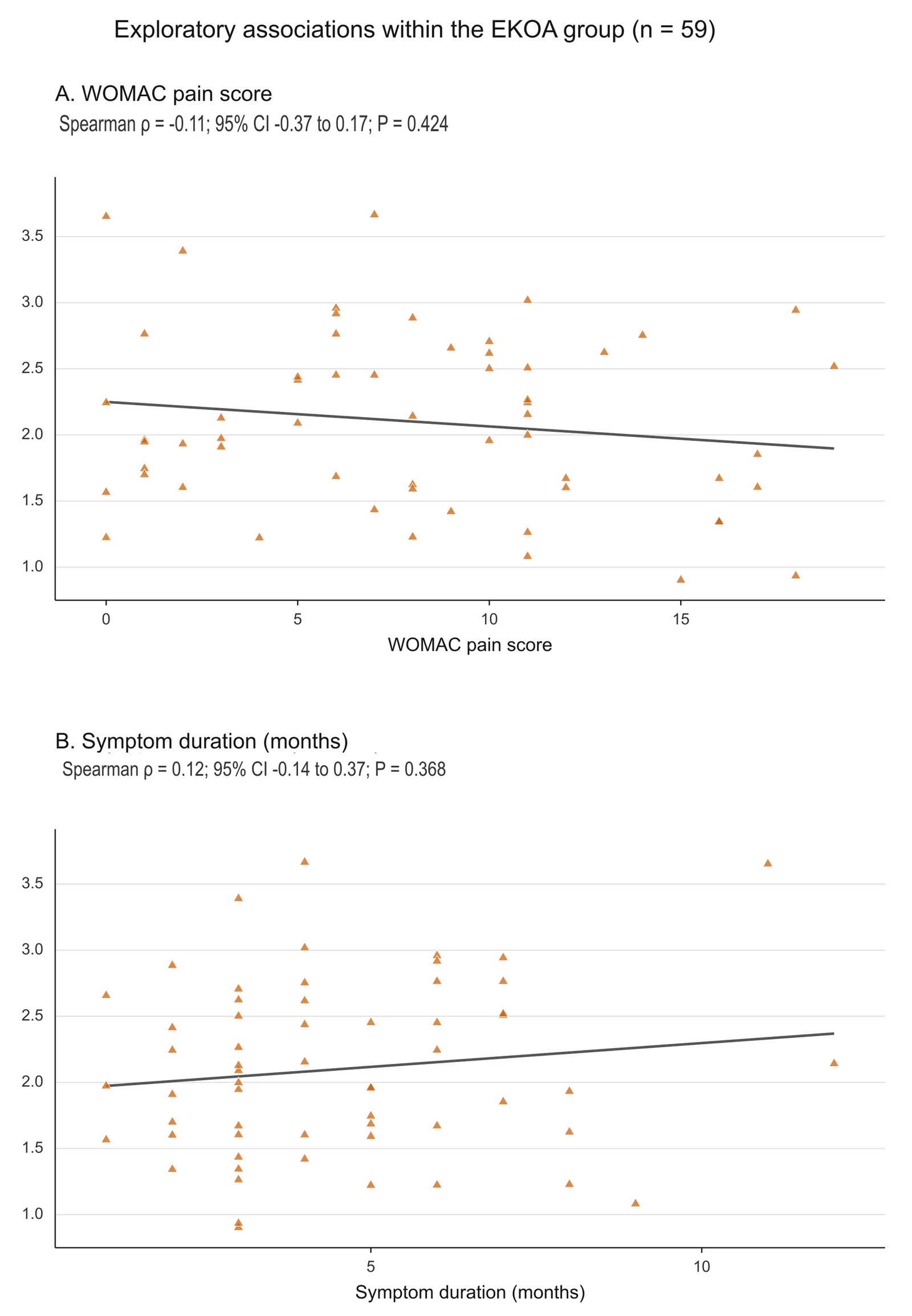
Exploratory clinical associations of the F ratio within the EKOA group. Panel (A) shows F ratio versus WOMAC pain score, and panel (B) shows F ratio versus symptom duration. Lines represent simple fitted trends; Spearman coefficients and 95% CIs are displayed. CI: confidence interval; EKOA: early knee osteoarthritis; WOMAC: Western Ontario and McMaster Universities Osteoarthritis Index

**Table 8:** Exploratory clinical associations of the F ratio. 95% CIs were obtained using 20,000 participant-level bootstrap resamples; all P-values were two-sided and unadjusted for multiplicity, as this was an exploratory analysis. K-L: Kellgren-Lawrence; WOMAC: Western Ontario and McMaster Universities Osteoarthritis Index; CI: confidence interval; EKOA: early knee osteoarthritis

Association	Group	n	Spearman ρ	CI lower bound	CI upper bound	P-value
F ratio vs K-L grade	Healthy control	54	-0.35	-0.579	-0.084	0.01
F ratio vs K-L grade	EKOA	59	-0.121	-0.4	0.182	0.361
F ratio vs WOMAC pain	EKOA	59	-0.106	-0.368	0.173	0.424
F ratio vs symptom duration	EKOA	59	0.119	-0.143	0.368	0.368
F ratio vs superficial cartilage morphology	EKOA	59	-0.231	-0.492	0.066	0.079
K-L vs superficial cartilage morphology	EKOA	59	0.379	0.121	0.595	0.003

In EKOA, the correlation between F ratio and superficial cartilage morphology grade was -0.231 (95% CI -0.492 to 0.066; P = 0.079). K-L grade correlated positively with superficial cartilage morphology grade (Spearman rho = 0.379; 95% CI 0.121 to 0.595; P = 0.003).

ROC analysis and internal validation

Using negative F ratio as the score for EKOA, the apparent AUC was 0.766 (bootstrap 95% CI 0.674 to 0.851). The within-sample Youden cutoff for the F ratio was 2.264, with sensitivity of 62.7% and specificity of 85.2%. After 2,000 bootstrap resamples, the optimism-corrected AUC was 0.765, and the corrected Youden index was 0.432 (Figure 7; Table 9).

**Figure 7:**
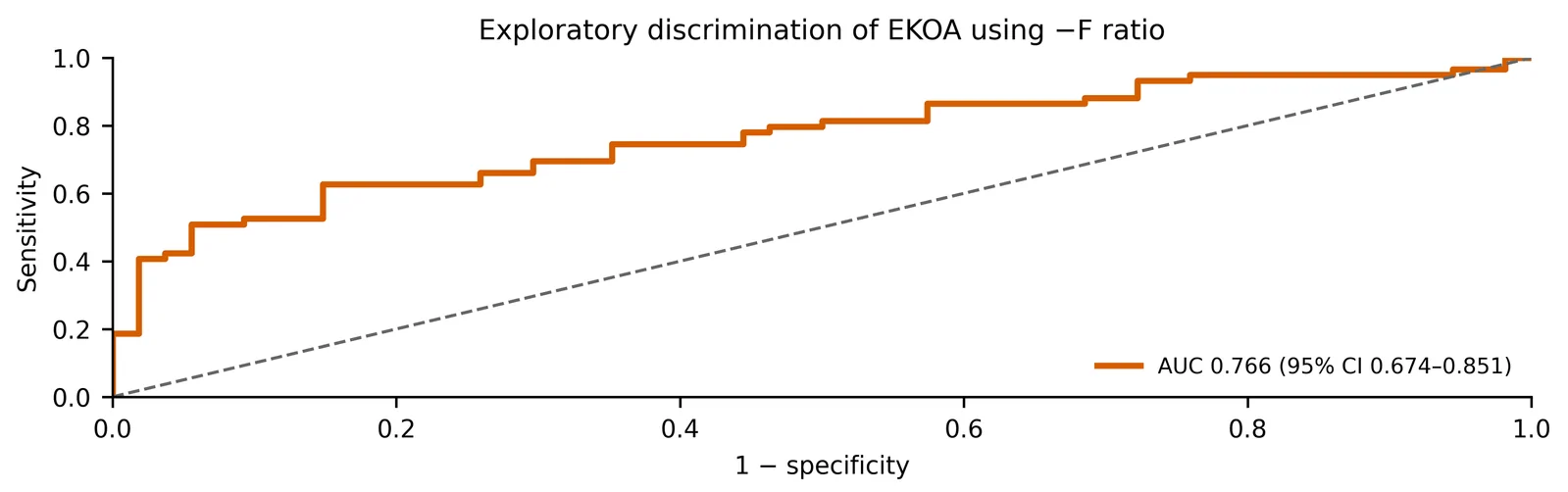
Exploratory ROC curve for discrimination of EKOA using the F ratio. ROC: receiver operating characteristic; EKOA: early knee osteoarthritis; AUC: area under the curve; CI: confidence interval

**Table 9:** ROC analysis and internal validation of the F ratio. -F was used as the score in the EKOA direction; lower F values provide greater support for EKOA. All results are exploratory within-sample estimates, without independent external validation. AUC: area under the curve; ROC: receiver operating characteristic; CI: 95% confidence interval; EKOA: early knee osteoarthritis

Measure	Estimate	CI lower bound	CI upper bound	Method
Apparent AUC	0.766	0.674	0.851	20,000 times stratified bootstrap
Within-sample F cutoff	2.264	Not estimated	Not estimated	Youden criterion
Sensitivity	0.627	0.424	0.814	Cutoff reselected at each resample
Specificity	0.852	0.667	1.0	Cutoff reselected at each resample
Optimism-corrected AUC	0.765	Not estimated	Not estimated	2,000 times bootstrap
Optimism-corrected Youden index	0.432	Not estimated	Not estimated	2,000 times bootstrap

Exploratory ROC results are shown in Figure 7 and Table 9: apparent AUC = 0.766 (95% CI 0.674-0.851), within-sample F cutoff = 2.264, sensitivity 62.7% (95% CI 42.4%-81.4%), specificity 85.2% (95% CI 66.7%-100.0%), and bootstrap optimism-corrected AUC = 0.765. This cutoff has not undergone external validation.

Exploratory decomposition and location analyses

After adjustment for age, sex, side, and BMI, group coefficients for log(F), log(N), and log(B) were -0.253, -0.162, and 0.091, respectively. The relative contribution of the numerator side was 63.9% (bootstrap 95% CI 34.0% to 95.6%), and that of the denominator side was 36.1% (95% CI 4.4% to 66.0%). The test for unequal contributions was not statistically significant (P = 0.220). These estimates have wide intervals and are interpreted as post hoc exploratory results.

In the three-location mixed-effects models, unadjusted group-by-location interaction P-values were 0.043 for F ratio, <0.001 for cartilage-plus-OCJ thickness, and 0.001 for OCJ thickness. Interactions for deep cartilage and subchondral bone plate thickness did not show clear statistical evidence. Correlations between imaging residuals and WOMAC pain score, symptom duration, and K-L grade within the EKOA group used unadjusted P-values and are interpreted as exploratory (Table 10).

**Table 10:** Mixed-effects analyses across three locations. Locations 1-3 were the prespecified medial, central, and lateral locations, respectively; all P-values were two-sided. Within-location comparisons used mixed-effects models, and q is the BH-FDR-adjusted value across the three locations for the same measure; group-by-location P-values are unadjusted and exploratory. GMR: EKOA/healthy-control geometric mean ratio; N = S + D + C; D: deep cartilage; C: OCJ; B: subchondral bone plate; CI: confidence interval; EKOA: early knee osteoarthritis; BH-FDR: Benjamini-Hochberg false discovery rate

Measure	Location	GMR	CI lower bound	CI upper bound	P-value	Within-subject rho	Group-by-location P	q-value
F	1	0.758	0.681	0.844	<0.001	0.86	0.043	<0.001
F	2	0.761	0.683	0.848	<0.001	0.86	0.043	<0.001
F	3	0.807	0.725	0.899	<0.001	0.86	0.043	<0.001
N	1	0.838	0.764	0.92	<0.001	0.945	<0.001	<0.001
N	2	0.833	0.759	0.914	<0.001	0.945	<0.001	<0.001
N	3	0.878	0.8	0.964	0.007	0.945	<0.001	0.007
D	1	1.05	0.928	1.187	0.44	0.915	0.083	0.44
D	2	1.059	0.937	1.198	0.358	0.915	0.083	0.44
D	3	1.106	0.978	1.251	0.11	0.915	0.083	0.33
C	1	0.988	0.887	1.1	0.82	0.905	0.001	0.82
C	2	0.981	0.881	1.092	0.725	0.905	0.001	0.82
C	3	1.057	0.95	1.177	0.31	0.905	0.001	0.82
B	1	1.106	1.008	1.214	0.034	0.865	0.789	0.074
B	2	1.095	0.998	1.201	0.057	0.865	0.789	0.074
B	3	1.088	0.992	1.194	0.074	0.865	0.789	0.074

Measurement reproducibility

The 23 repeat-measurement participants represented 20.4% of all 113 participants. Inter-reader ICCs for six continuous measures were 0.988-0.998, and intra-reader ICCs were 0.990-0.998; F ratio inter-reader ICC = 0.988 (95% CI 0.960-1.000) and intra-reader ICC = 0.993 (95% CI 0.980-1.000). Full ICC, kappa, raw-agreement, and Bland-Altman results are shown in Tables 11-13 and Figures 8-12.

**Table 11:** Measurement reproducibility of the F ratio. Differences were reader 2 first measurement minus reader 1 first measurement (inter-reader) or reader 1 second measurement minus first measurement (intra-reader). Inter-reader ICC used a two-way random-effects, absolute-agreement, single-measurement model; intra-reader ICC used a two-way mixed-effects, absolute-agreement, single-measurement model. LoA: Bland-Altman 95% limits of agreement; CI: 95% confidence interval; ICC: intraclass correlation coefficient

Measure	Comparison	n	ICC(A,1)	ICC CI lower bound	ICC CI upper bound	Bias	Lower LoA	Upper LoA
F ratio	Inter-reader	23	0.988	0.960	1.000	0.056	-0.132	0.244
F ratio	Intra-reader	23	0.993	0.980	1.000	-0.007	-0.170	0.155

**Table 12:** Reproducibility of categorical and ordinal measures (random sample, 23/113 (20.4%)). Ordinal measures used quadratically weighted Cohen kappa; binary measures used unweighted Cohen kappa. The 95% CIs were obtained from 10,000 participant-level bootstrap resamples with fixed random seed 20260904. Percentages use 23 valid pairs as the denominator. OCJ: osteochondral junction; CI: confidence interval

Measure	Comparison	n	Kappa type	κ	CI lower bound	CI upper bound	Agreement (%)
Superficial cartilage morphology	Inter-reader	23	Quadratically weighted Cohen kappa	0.871	0.617	1.000	87.0
OCJ morphology	Inter-reader	23	Quadratically weighted Cohen kappa	0.942	0.779	1.000	87.0
Marginal micro-osteophyte	Inter-reader	23	Unweighted Cohen kappa	0.817	0.534	1.000	91.3
Meniscal grade	Inter-reader	23	Quadratically weighted Cohen kappa	0.924	0.774	1.000	87.0
Ligament insertion grade	Inter-reader	23	Quadratically weighted Cohen kappa	0.923	0.785	1.000	87.0
Low-signal lesion severity (none < linear < patchy)	Inter-reader	23	Quadratically weighted Cohen kappa	0.775	0.452	1.000	87.0
Any low-signal lesion	Inter-reader	23	Unweighted Cohen kappa	0.826	0.553	1.000	91.3
Superficial cartilage morphology	Intra-reader	23	Quadratically weighted Cohen kappa	0.879	0.674	1.000	87.0
OCJ morphology	Intra-reader	23	Quadratically weighted Cohen kappa	0.944	0.787	1.000	87.0
Marginal micro-osteophyte	Intra-reader	23	Unweighted Cohen kappa	0.817	0.534	1.000	91.3
Meniscal grade	Intra-reader	23	Quadratically weighted Cohen kappa	0.920	0.790	1.000	87.0
Ligament insertion grade	Intra-reader	23	Quadratically weighted Cohen kappa	0.927	0.800	1.000	87.0
Low-signal lesion severity (none < linear < patchy)	Intra-reader	23	Quadratically weighted Cohen kappa	0.775	0.450	1.000	87.0
Any low-signal lesion	Intra-reader	23	Unweighted Cohen kappa	0.826	0.553	1.000	91.3

**Table 13:** Reproducibility of continuous measures (random sample, 23/113 (20.4%)). Inter-reader differences were reader 2 first measurement minus reader 1 first measurement; intra-reader differences were reader 1 second measurement minus first measurement. Proportional-bias P-values were from two-sided linear regressions of differences on means. ICC model definitions are as in Table 11. LoA: 95% limits of agreement; OCJ: osteochondral junction; ICC: intraclass correlation coefficient; CI: confidence interval

Measure	Comparison	n	ICC(A,1)	ICC CI lower bound	ICC CI upper bound	Bias	Lower LoA	Upper LoA	Proportional-bias P
Superficial cartilage	Inter-reader	23	0.998	0.990	1.000	0.012	-0.054	0.077	0.522
Deep cartilage	Inter-reader	23	0.993	0.980	1.000	0.011	-0.063	0.085	0.520
OCJ	Inter-reader	23	0.996	0.990	1.000	0.011	-0.049	0.071	0.311
Subchondral bone plate	Inter-reader	23	0.993	0.980	1.000	-0.017	-0.113	0.079	0.299
Total osteochondral-unit thickness	Inter-reader	23	0.996	0.990	1.000	0.033	-0.076	0.142	0.303
F ratio	Inter-reader	23	0.988	0.960	1.000	0.056	-0.132	0.244	0.064
Superficial cartilage	Intra-reader	23	0.998	0.990	1.000	-0.018	-0.067	0.030	0.016
Deep cartilage	Intra-reader	23	0.992	0.980	1.000	0.005	-0.078	0.087	0.133
OCJ	Intra-reader	23	0.995	0.990	1.000	0.009	-0.062	0.080	0.034
Subchondral bone plate	Intra-reader	23	0.990	0.980	1.000	-0.002	-0.122	0.117	0.419
Total osteochondral-unit thickness	Intra-reader	23	0.996	0.990	1.000	-0.005	-0.132	0.123	0.041
F ratio	Intra-reader	23	0.993	0.980	1.000	-0.007	-0.170	0.155	0.600

**Figure 8:**
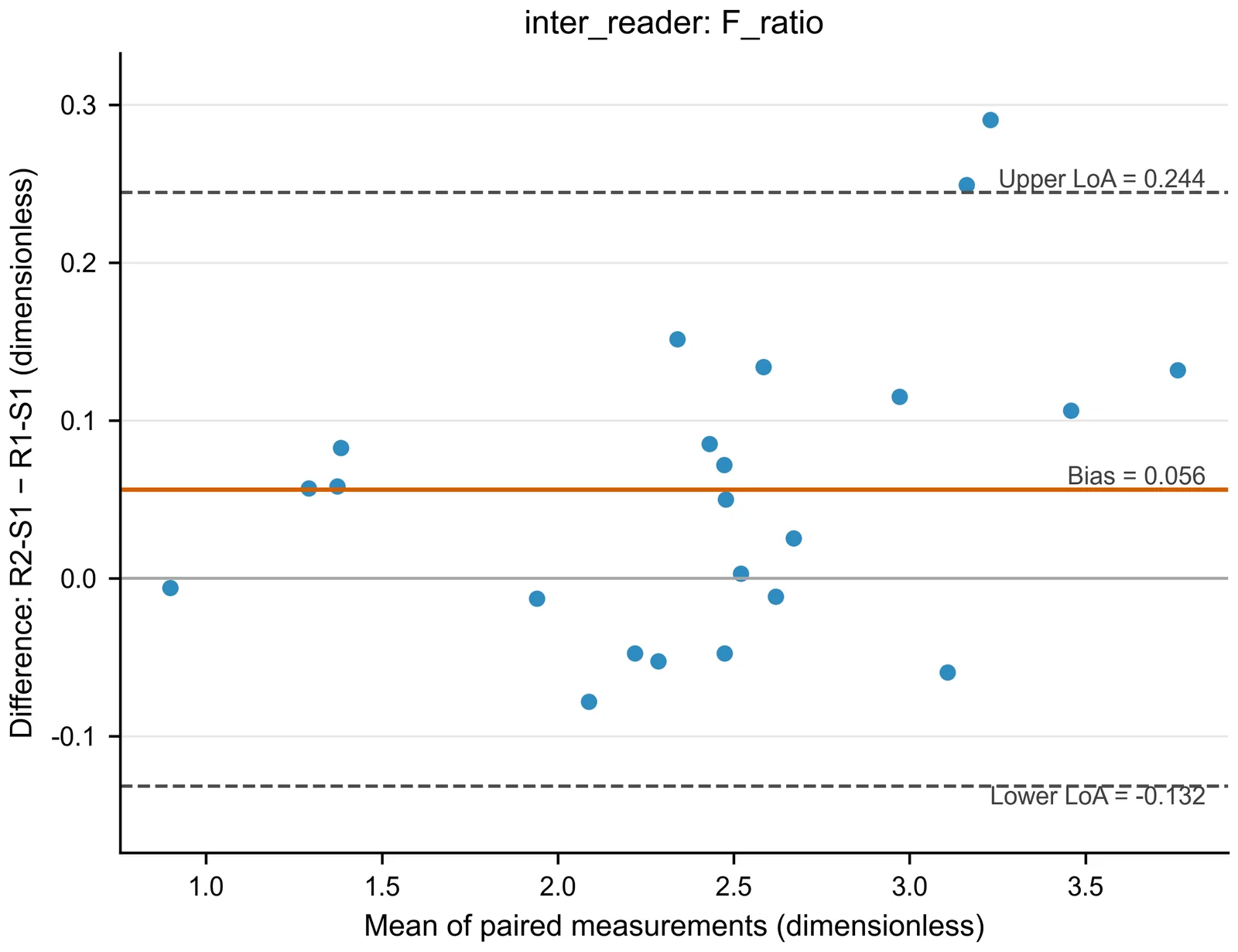
Inter-reader Bland-Altman agreement for the F ratio. This figure was generated from this study's original de-identified repeat-measurement data. LoA: limits of agreement

**Figure 9:**
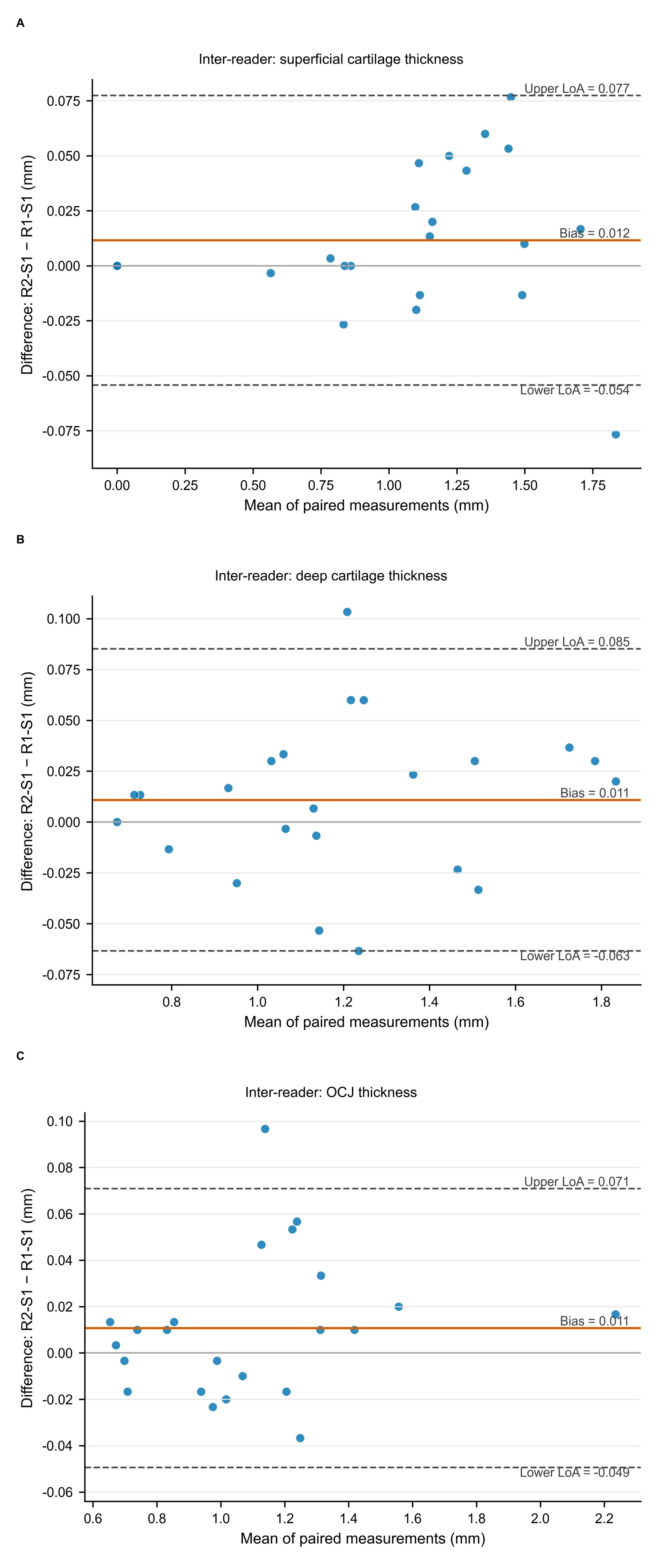
(A-C) Bland-Altman plots of inter-reader superficial-cartilage, deep-cartilage, and OCJ thicknesses. OCJ: osteochondral junction; LoA: limits of agreement

**Figure 10:**
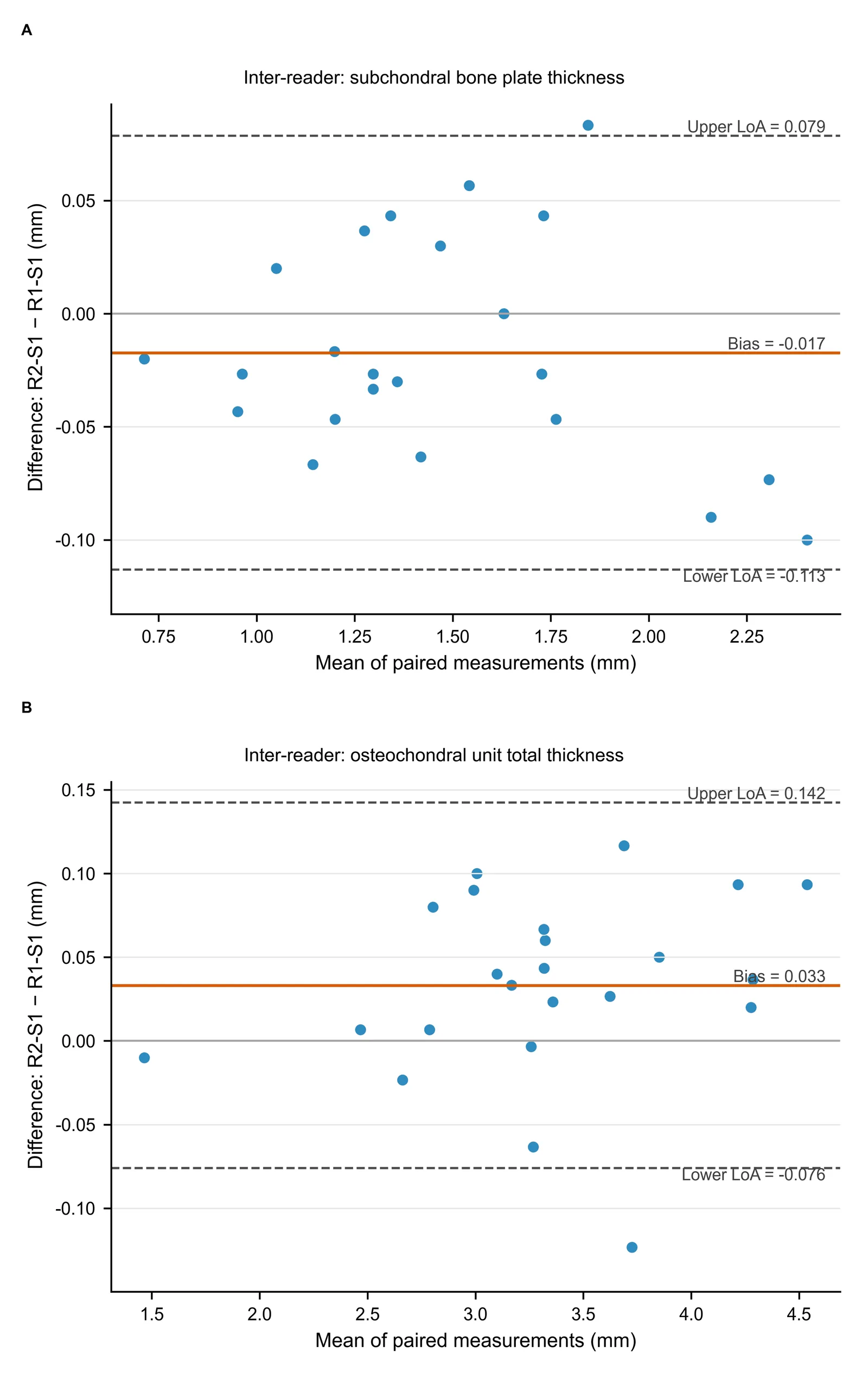
(A, B) Bland-Altman plots of inter-reader subchondral-bone-plate and total osteochondral-unit thicknesses. LoA: limits of agreement

**Figure 11:**
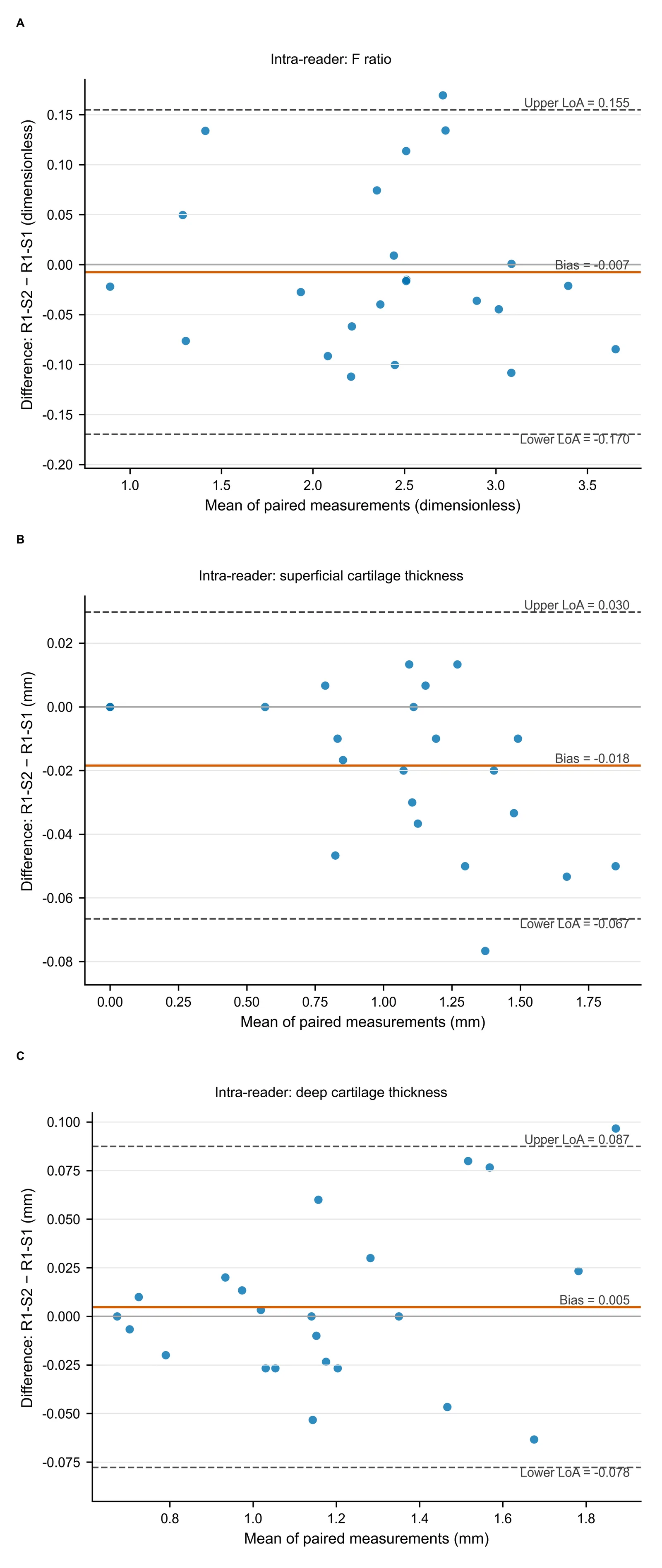
(A-C) Bland-Altman plots of intra-reader F ratio, superficial-cartilage, and deep-cartilage thicknesses. LoA: limits of agreement

**Figure 12:**
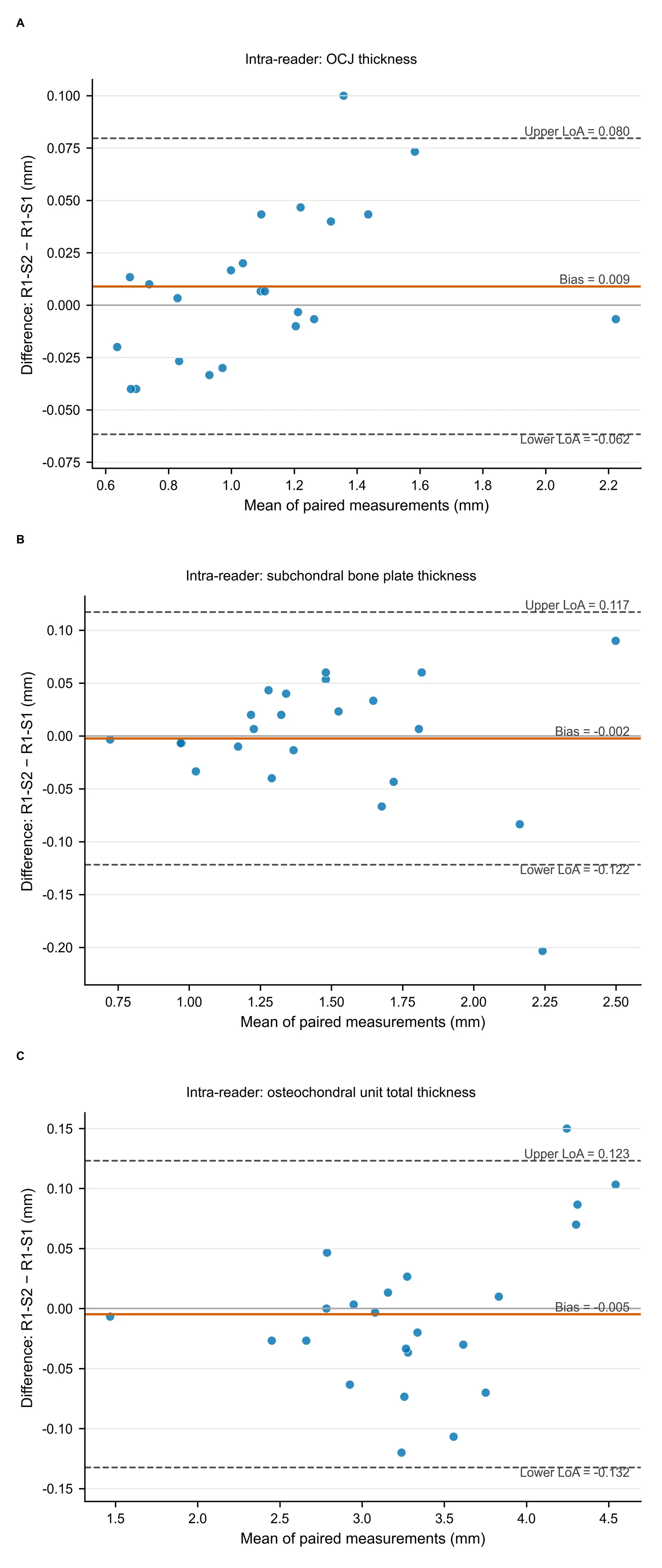
(A-C) Bland-Altman plots of intra-reader OCJ, subchondral-bone-plate, and total osteochondral-unit thicknesses. OCJ: osteochondral junction; LoA: limits of agreement

Intra-reader measurements of superficial cartilage, OCJ, and cartilage-plus-OCJ thickness showed proportional bias, with P-values of 0.016, 0.034, and 0.041 for the relation between measurement difference and mean, respectively. Supplementary analyses after logarithmic transformation of OCJ and total thickness yielded GMRs of 1.003 (ratio limits of agreement, 0.940 to 1.071) and 0.997 (0.963 to 1.032), respectively. Additional Bland-Altman plots are provided in Figure 9. The inter-reader and intra-reader kappa values, 95% CIs, and raw agreement rates for the categorical imaging features are shown in Table 12; the inter-reader and intra-reader ICCs, mean biases, 95% limits of agreement, and proportional bias test results for all continuous layer-thickness metrics are shown in Table 13.

## Discussion

Principal findings

In this clinically and radiologically defined EKOA population, the PETRA-derived osteochondral F ratio was lower than in healthy controls, and the difference remained stable after adjustment for age, sex, side, and BMI. Superficial cartilage thinning and non-identifiability were prominent accompanying findings, and OCJ morphology, meniscal score, and ligament-insertion score also differed between groups. F ratio showed high inter-reader and intra-reader reproducibility and a moderate level of within-sample discrimination after optimism correction. These findings support F ratio as a candidate measure of the osteochondral structural phenotype in EKOA, particularly for supplementary structural assessment in individuals with persistent knee pain and mild radiographic change.

Structural interpretation of F ratio and ROC findings

F ratio places cartilage-plus-OCJ thickness and adjacent bone plate thickness within a single measure, allowing their relative composition to be quantified. The lower F ratio in EKOA was consistent with thinner measurable superficial cartilage, non-identifiability of this layer in some cases, lower cartilage-plus-OCJ thickness, and greater subchondral bone plate thickness. Interpretation of the F ratio requires inspection of both numerator and denominator rather than attribution of the ratio to a single tissue.

The log-scale decomposition estimated contributions of 63.9% from the cartilage-plus-OCJ side and 36.1% from the bone plate side, providing a structural clue to the difference in F ratio. Because this decomposition follows the mathematical identity log(F) = log(N) - log(B), its estimates describe the contribution of measured values to the group difference and do not quantify pathologic proportions of cartilage degeneration or bone remodeling. F ratio, individual layer thicknesses, and morphological features should therefore be reported together to clarify the structural pattern underlying a low ratio.

Previous studies indicate that early osteoarthritic cartilage change does not necessarily present as uniform thinning. Some joints may first show focal cartilage swelling or thickening. Experimental osteoarthritis studies have shown that increased cartilage thickness in weight-bearing regions can precede clear surface erosion and subchondral remodeling. Proteoglycan loss, reduced collagen-network restraint, and increased tissue water content may underlie this pattern, so thickening may reflect early matrix injury rather than repair [17,18]. Later longitudinal work did not show consistently sustained thickening and instead found substantial variability in EKOA cartilage-thickness change, suggesting that swelling, stability, and thinning may coexist across individuals and regions [19]. In the region measured by PETRA, early swelling could increase apparent superficial or deep cartilage thickness before the subchondral bone plate thickens, leading to a transiently higher or normal F ratio. With progression, superficial cartilage thinning, focal loss, or non-identifiability together with subchondral bone plate thickening may lower the ratio. This stage-dependent, regional, and interindividual heterogeneity may contribute to overlap in F ratio distributions and to the moderate discrimination observed in this sample. This mechanistic interpretation requires longitudinal PETRA, site-matched thickness measurement, and histologic or quantitative compositional imaging for validation. The AUC 95% CI of 0.674 to 0.851 included 0.80, so the present data do not establish that the population AUC is below 0.80.

An advantage of the F ratio is that it considers changes in adjacent tissues within the same osteochondral unit. In patients with similar radiographic grades, it can be used to study internal osteochondral differences alongside whole-joint information such as meniscal extrusion and bone marrow lesions. Longitudinal Osteoarthritis Initiative data show that meniscal extrusion and bone marrow lesions are associated with incident or progressive KOA, supporting multistructural assessment that includes local osteochondral evaluation [20]. Dedicated validation is still needed to determine how resistant the F ratio is to differences in individual joint size and localization.

Clinical interpretation of superficial cartilage non-identifiability

Superficial cartilage was non-identifiable on PETRA in 27.1% of EKOA knees and in no control knees, making this feature worthy of focused review as a local structural abnormality. Ex vivo PETRA-fat-suppressed histologic work supports UTE imaging of non-calcified cartilage and cartilage injury, whereas this study used in vivo acquisition with different sequence conditions and spatial resolution [11]. We therefore defined this feature as non-identifiability of an imaging layer and retained its morphologic and quantitative information through separate binary analysis and the prespecified zero-coding rule.

In patients with persistent knee pain and mild radiographic change, this feature should prompt review of local cartilage continuity, the adjacent bone plate, and conventional MRI findings, together with history, examination, and load-related symptoms. The meaning of an imaging abnormality depends on the clinical context. The Framingham study found that middle-aged and older adults without radiographic KOA frequently have MRI structural abnormalities, including in painless knees [21]. Reports should therefore use descriptive wording, such as "non-identifiable superficial cartilage band on PETRA," while clinical attribution and the extent of injury are determined through integrated multimodal assessment.

Relation to previous short-T2 imaging evidence

Previous short-T2 imaging studies provide compositional evidence relevant to osteochondral unit assessment. Ex vivo UTE magnetization-transfer work found that magnetization transfer ratio was associated with histologic cartilage degeneration grade [22]. Paired preoperative in vivo imaging and arthroplasty-specimen studies also linked UTE measures with histologic and biochemical changes [23]. Another in vivo OCJ study found lower macromolecular fraction and magnetization transfer ratio in K-L grade 2 to 3 knees than in K-L grade 0 to 1 knees [24]. These studies covered different disease spectra and tissue references; the PETRA thickness ratio in the present study adds structural-scale information.

Deep cartilage and OCJ mean thicknesses did not differ significantly between groups, whereas OCJ morphology differed, indicating that mean thickness and local interface morphology should be interpreted together. A single time-point comparison cannot establish the sequence of these changes. Ex vivo quantitative UTE imaging has mapped OCJ T1, T1rho, T2*, and macromolecular fraction, providing a technical basis for integrating structural measurements with compositional imaging [25]. Building on the exploratory location analyses, subsequent studies can test associations among local thickness ratios, morphology, compositional change, weight-bearing region, knee alignment, and meniscal extrusion.

Position in the clinical workflow

The apparent AUC and optimism-corrected AUC both indicated moderate within-sample discrimination. The exploratory cutoff of 2.264 had sensitivity of 62.7% and specificity of 85.2%, indicating that a lower F ratio identified structural abnormalities in some participants with EKOA while many cases remained above that threshold. In routine practice, knee pain has multiple causes, and accuracy estimated from a case-control comparison depends on the disease spectrum and reference standard. Diagnostic-accuracy studies should specify the intended population and the position of the test in the clinical pathway [26]. F ratio is therefore best regarded at present as a supplementary structural measure after conventional evaluation. Its clinical threshold requires validation in consecutively presenting symptomatic populations.

Clinically, PETRA is better suited as a supplementary structural assessment tool alongside conventional radiography and MRI. In patients with persistent knee pain but mild weight-bearing radiographic changes (K-L grades 0-2), PETRA may be used, in conjunction with history, examination, and conventional MRI, to assess the short-T2 osteochondral interface, superficial-cartilage continuity, and subchondral bone plate and thereby document local structural changes not readily shown on conventional sequences. At present, the F ratio or a within-sample cutoff should not be used alone to diagnose EKOA or determine treatment.

PETRA added only 2 minutes and 19 s and produced three-dimensional images that could be reconstructed in multiple planes, supporting its feasibility within a routine knee MRI protocol. In research use, symptoms, examination findings, weight-bearing radiographs, and conventional MRI can first inform integrated assessment; F ratio, superficial cartilage identifiability, and OCJ morphology can then provide trackable osteochondral descriptors. Treatment decisions should continue to be based on symptoms, function, body weight, and comorbidities. Osteoarthritis Research Society International (OARSI) guidelines identify patient education, structured exercise, and weight management when appropriate as core KOA interventions [27], and the IDEA randomized trial supports intensive diet-induced weight loss combined with exercise for overweight or obese adults with KOA [28]. Whether PETRA improves stratification or treatment selection beyond established management requires direct evaluation with prespecified decision outcomes.

Spearman correlations of F ratio with WOMAC pain score and symptom duration in EKOA were close to zero, with CIs crossing zero. The structural difference measured in this study therefore did not show a clear relation to symptom severity. Longitudinal MRI studies have linked changes in bone marrow lesions and synovitis with fluctuations in knee pain, indicating that pain assessment should consider multiple tissue sources [29]. PETRA structural information and patient-reported outcomes such as WOMAC should be recorded in parallel to describe tissue status and symptom burden separately. The present findings support further investigation of their complementary roles.

Measurement reproducibility and implementation conditions

Under the standardized localization, reconstruction, and layer-identification protocol, inter-reader and intra-reader ICCs for F ratio were 0.988 and 0.993, respectively, supporting good reproducibility of manual measurement under the conditions of this study. Bland-Altman results quantify the range of individual measurement error, and some thin-layer measures showed proportional bias. High ICCs reflect measurement reliability in this sample; assessment of individual longitudinal change also requires limits of agreement and repeat-scan reproducibility.

Spatial resolution is a key condition for clinical translation. The acquired voxel size was approximately 1.041 mm, and the thickness of some bands approached this scale. Apparent thickness and identifiability may therefore be affected by partial-volume effects, local signal contrast, and reconstruction direction. Future implementation should standardize acquisition, reconstruction, display, and localization; conduct repeat-scan and cross-platform validation; and define the smallest detectable change. These steps are required before reader measurement reproducibility can be translated into reliable longitudinal structural assessment.

Limitations and future validation

This single-center case-control study evaluated structural differences at enrollment within the present PETRA protocol and the included EKOA and healthy control populations. Differences in symptoms and K-L-grade distributions between groups may influence within-sample discrimination; independent validation should prioritize clinically presenting patients with comparable symptoms and radiographic grades. Individual layer thicknesses and superficial cartilage non-identifiability lacked a site-matched histologic or arthroscopic reference. The prespecified rule assigning 0 mm to a non-identifiable layer can also influence F ratio and requires evaluation with higher-resolution imaging and alternative encoding strategies. Reader repeat measurements did not capture repeat-scan, cross-platform, or multicenter error. Exploratory correlation, decomposition, and location analyses require confirmation in larger samples. Independent clinical cohorts, multicenter repeat scanning, histologic reference, and longitudinal follow-up are needed to establish measurement accuracy and external applicability of F ratio and to determine whether PETRA adds information to routine knee MRI for clinical stratification and follow-up.

Future multicenter longitudinal studies using fixed acquisition and measurement protocols should examine associations of F ratio with cartilage loss, radiographic progression, persistent pain, and functional decline. Clinical value should be assessed by comparing clinical information plus conventional imaging with clinical information plus conventional imaging and PETRA. In addition to discrimination, these studies should report calibration and, when decision thresholds and downstream actions are defined, evaluate net benefit with decision-curve analysis [30]. Establishing measurement error, detectable change, and patient-relevant outcomes will clarify when PETRA measures provide information that can alter management or support trial stratification.

The next step should be preregistered external validation across different scanners, coils, spatial resolutions, and reconstruction platforms, and testing of associations between the F ratio and persistent pain, cartilage loss, and imaging progression in longitudinal cohorts. Clinical thresholds should be locked in an independent sample before calibration and net clinical benefit is evaluated.

## Conclusions

This study shows that clinical 3.0 T PETRA can depict osteochondral layer bands that are not easily fully assessed on conventional MRI with a short additional scan time. The F ratio was lower in EKOA than in healthy controls, and this difference was consistent in non-parametric testing, covariate-adjusted analyses, and sensitivity analyses; it was accompanied by superficial-cartilage thinning or absent structure, subchondral-bone-plate thickening, and lower total osteochondral-unit thickness. PETRA and the F ratio may provide supplementary structural information for patients with persistent knee pain and mild radiographic changes, but cannot replace integrated clinical assessment, and within-sample cutoffs cannot be used directly as diagnostic thresholds. Their reproducibility, prognostic value, and cross-platform applicability require independent, multicenter, longitudinal validation.
